# Mouse-adapted SARS-CoV-2 Omicron BA.5 infection induces post-acute lung fibrosis in BALB/c mice

**DOI:** 10.1128/jvi.01406-25

**Published:** 2025-11-06

**Authors:** John M. Powers, Sarah R. Leist, Naveenchandra Suryadevara, Seth J. Zost, Elad Binshtein, Anfal Abdelgadir, Michael L. Mallory, Caitlin E. Edwards, Kendra L. Gully, Miranda L. Hubbard, Mark R. Zweigart, Alexis B. Bailey, Timothy P. Sheahan, James E. Crowe, Stephanie A. Montgomery, Jack R. Harkema, Ralph S. Baric

**Affiliations:** 1 Department of Epidemiology, Gillings School of Global Public Health, University of North Carolina at Chapel Hill 41474, Chapel Hill, North Carolina, USA; 2 Vanderbilt Center for Antibody Therapeutics, Vanderbilt University Medical Center, Nashville, Tennessee, USA; 3 Department of Microbiology and Immunology, University of North Carolina at Chapel Hill 318275, Chapel Hill, North Carolina, USA; 4 Department of Pathology, Microbiology, and Immunology, Vanderbilt University Medical Center 204907, Nashville, Tennessee, USA; 5 Department of Pediatrics, Vanderbilt University Medical Center 12328, Nashville, Tennessee, USA; 6 Lineberger Comprehensive Cancer Center, University of North Carolina at Chapel Hill 169113, Chapel Hill, North Carolina, USA; 7 Department of Pathology and Laboratory Medicine, University of North Carolina at Chapel Hill 196290, Chapel Hill, North Carolina, USA; 8 Department of Pathobiology and Diagnostic Investigation, Michigan State University 573378, East Lansing, Michigan, USA; St Jude Children's Research Hospital, Memphis, Tennessee, USA

**Keywords:** sarbecovirus, fibrotic lung disease, mouse models, monoclonal antibodies, SARS-CoV-2 Omicron BA.5

## Abstract

Following severe acute respiratory syndrome coronavirus 2 (SARS-CoV-2) Omicron BA.1, subsequent Omicron sub-lineages have continued to emerge, challenging the development of intervention and prevention strategies, including monoclonal antibodies and vaccines. To better understand the pathogenic effects caused by Omicron BA.5 infection, we developed a mouse-adapted virus with overt disease burden in BALB/c mice. Acute disease was characterized by significant weight loss and lung dysfunction following high-dose challenges. In survivor animals that were followed through 107 days post-infection, subpleural fibrosis with associated tertiary lymphoid structures was noted. Serum from these mice demonstrated potent neutralization against BA.5, with substantially reduced neutralization titers against early epidemic, zoonotic, and more recent contemporary XBB.1.5 variants. Intervention with pre-clinical monoclonal antibodies revealed that robust protection from BA.5-induced lung disease was possible after prophylactic administration. Together, this model enables the investigation of therapeutic approaches for both acute and post-acute sequelae of COVID-19.

**IMPORTANCE:**

To best combat the evolving landscape of SARS-CoV-2 variants of interest and variants of concern, the development of effective small animal models is of critical importance. Herein, we describe the development of a model system in BALB/c mice to study the effects of SARS-CoV-2 BA.5 S gene in both acute and chronic disease manifestations. Intriguingly, we determined that fibrotic lung disease with tertiary lymphoid structures was a prominent feature in the lungs of mice that survived through the acute phase of infection. This is a prominent concern in human patients that survive the initial infection insult. As such, and most critically, the model system presented here provides researchers with an effective pathway in which long COVID manifestations and potential interventions can be studied.

## INTRODUCTION

Severe acute respiratory syndrome coronavirus 2 (SARS-CoV-2) virions contain an approximately 30 kb positive-sense single-stranded viral genome that encodes numerous structural proteins (spike, membrane, envelope, and nucleocapsid), non-structural proteins (nsp1–16), and several accessory genes (1, 2). Following its emergence in late 2019, SARS-CoV-2 rapidly evolved into numerous variants of interest and variants of concern (VOC). The Omicron B.1.1.529 VOC (BA.1) was of significant interest because it encoded a substantial number of amino acid changes within the main antigenic site, the spike gene (S), especially within the receptor-binding domain (RBD), and the receptor-binding motif (RBM). BA.5, which emerged in early 2022, is a descendant lineage of Omicron that contained additional alterations within the RBD/RBM protein sequence. Due to the role of the spike protein (S) in cellular attachment to angiotensin-converting enzyme 2 (ACE2) and subsequent viral entry following its cleavage by transmembrane protease serine 2 (TMPRSS2), the expanded spike variation was associated with enhanced receptor binding, antibody evasion, and reduced efficacy of both natural and vaccine-induced immunity (3). Amino acid mutations in these domains enabled escape from neutralizing antibodies (4–9).

Inconsistent results in mouse models using BA.5 isolates were observed by different research groups regarding the degree of pathogenicity as compared to BA.1 or previous ancestral viruses (10, 11). However, in both cases, disease severity was overall attenuated compared to early VOCs, like Alpha and Delta, likely mitigated by differences in the clinical isolate, inoculum dose, mouse strain, sex, and age of animals (12–14). Consequently, we generated recombinant SARS-CoV-2 viruses to study the effect of the BA.5 S protein in pathogenicity. As such, we utilized a reverse genetics infectious clone system in order to replace the spike gene of our previously described MA10 virus with the spike gene sequence from BA.5 (designated BA.5 MA) (15–17). In parallel, we also recovered a derivative strain without mouse adaptations in which the viral ORF7a was replaced with a gene encoding the reporter protein nanoluciferase (nLuc) to be used as an indicator virus in live-virus neutralization assays (BA.5 nLuc) (15–20). BA.5 MA infection in young (14- to 16-week-old) or aged (10- to 12-month-old) female BALB/c mice caused severe diffuse alveolar damage with accompanying mortality and high virus titers in the lungs and nasal turbinates, which was significantly attenuated by prophylactic administration of monoclonal antibodies (mAbs). In contrast, we also produced viruses expressing the BA.2 Omicron S protein on the same viral backbones to be used in *in vivo* and *in vitro* assays as were performed with BA.5. Infection of aged female BALB/c mice with BA.2 MA failed to produce overt disease symptoms such as weight loss and mortality but did replicate to high viral titers in both the lungs and nasal turbinates.

SARS-CoV-2 symptomatic or asymptomatic infection may progress to post-acute sequelae of SARS-CoV-2 (PASC), a frequent chronic disease syndrome that includes a continuous, relapsing and remitting, or progressive disease state that affects one or more organ systems and lasts for weeks to years in about ~10% of SARS-CoV-2 survivors (21–23). In the respiratory tract, shortness of breath, cough, persistent fatigue, post-exertional malaise, and diagnosable conditions like interstitial lung disease and hypoxemia are among the most frequent chronic disease phenotypes. Omicron-related infections are reported to cause less frequent PASC disease in humans as compared to ancestral VOCs (24). To begin to dissect potential virus-centric differences in PASC disease potential, we first sought to develop Omicron-based models of acute and post-acute lung pathogenesis using mouse-adapted SARS-CoV-2 bearing BA.5 or BA.2 S proteins. We show that S is a major driver of acute pathogenesis and that severe acute disease can drive post-acute lung sequelae. The humoral immune response was dominated by homotypic responses, and the magnitude of heterotypic neutralizing responses correlated with genetic relatedness to Omicron. We also demonstrate these models can be used to evaluate antiviral therapies. Altogether, these models can be leveraged to study pathogenic mechanisms of acute and long COVID and whether pre-existing long COVID conditions impact acute/chronic disease potential for future SARS-CoV-2 VOC or even other respiratory virus infections.

## RESULTS

### Recovery of Omicron recombinant viruses

**Fig 1:**
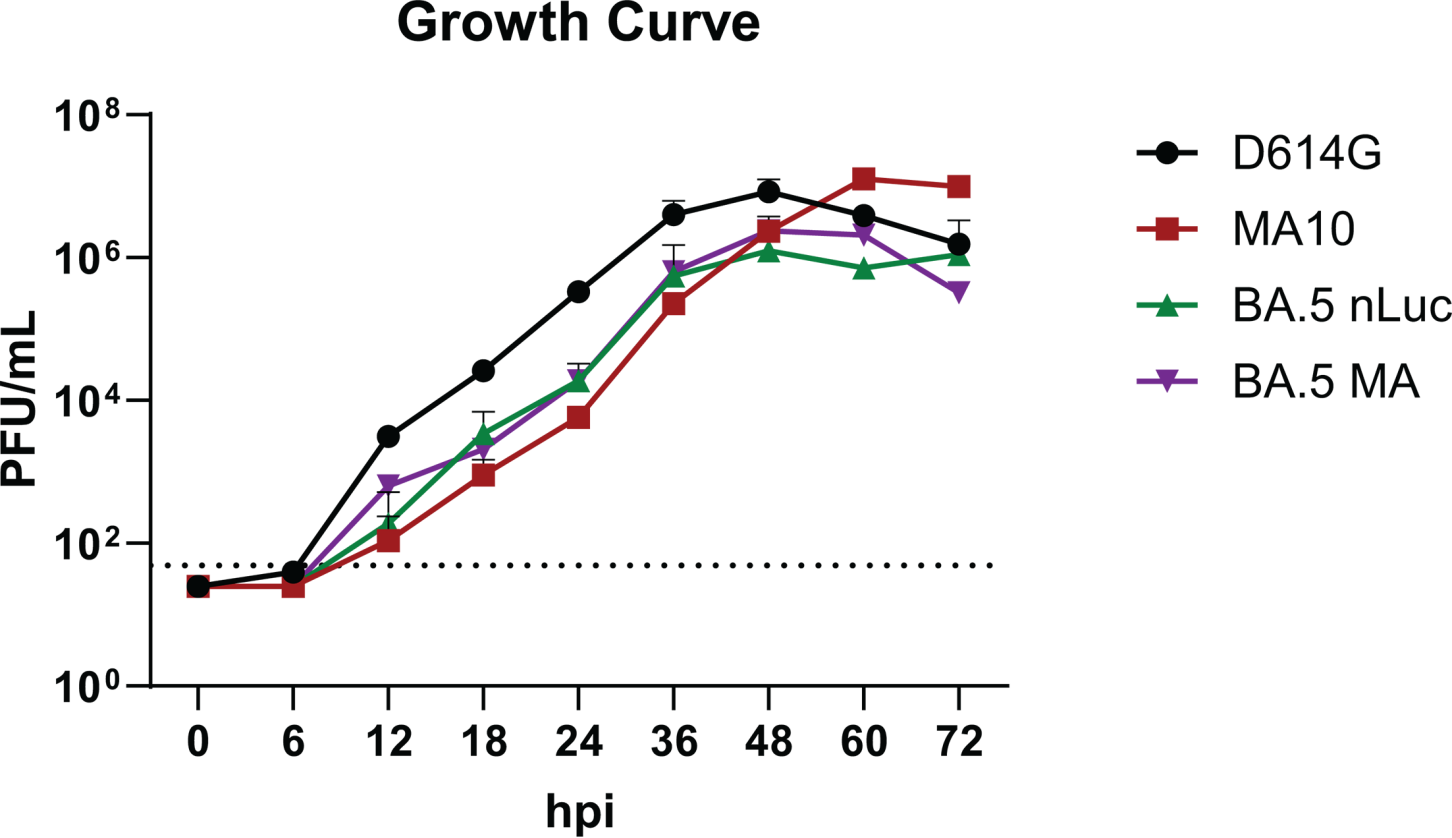
Growth curve analysis identifies replication competency of BA.5 MA and nLuc. Growth dynamics for the recombinant BA.5 MA and BA.5 nLuc viruses were compared to ancestral counterparts (D614G nLuc and MA10) to assess for replication competency and equivalent or attenuated replication. Maximal virus titers were reached between 48 and 60 hours post-infection with an MOI of 0.001. Both parental viruses achieved maximal titers ~10-fold higher than those of the recombinant BA.5 viruses.

Using reverse genetics, we generated a mouse-adapted SARS-CoV-2 MA10 recombinant bearing the BA.2 or BA.5 subvariant spike (BA.2 MA and BA.5 MA) and a related reporter virus where we replaced ORF7a with nanoluciferase (BA.2 nLuc and BA.5 nLuc) (15–17). Genome schematics of the infectious clone constructs are shown in Fig. S1. To assess replication kinetics, the growth of the two BA.5 recombinant viruses was compared to that of the ancestral strains SARS-CoV-2 MA10 and D614G nLuc, at an MOI of 0.001 on Vero E6 cells. The growth kinetics of BA.5 MA and BA.5 nLuc were the same, indicating that replication was not impacted by the inclusion of the reporter gene. The ancestral pandemic strain (SARS-CoV-2 D614G) had a growth advantage over both BA.5 viruses at most times assessed (Fig. 1). Importantly, parental SARS-CoV-2 MA10 virus grew similarly to both BA.5 viruses early in the kinetic, yet parental MA10 titers exceeded those of BA.5 viruses at later timepoints (Fig. 1). Altogether, these data demonstrate that our BA.5. spike recombinant viruses grew efficiently in Vero cells.

### Pathological features of BA.5 MA infection in young mice

**Fig 2:**
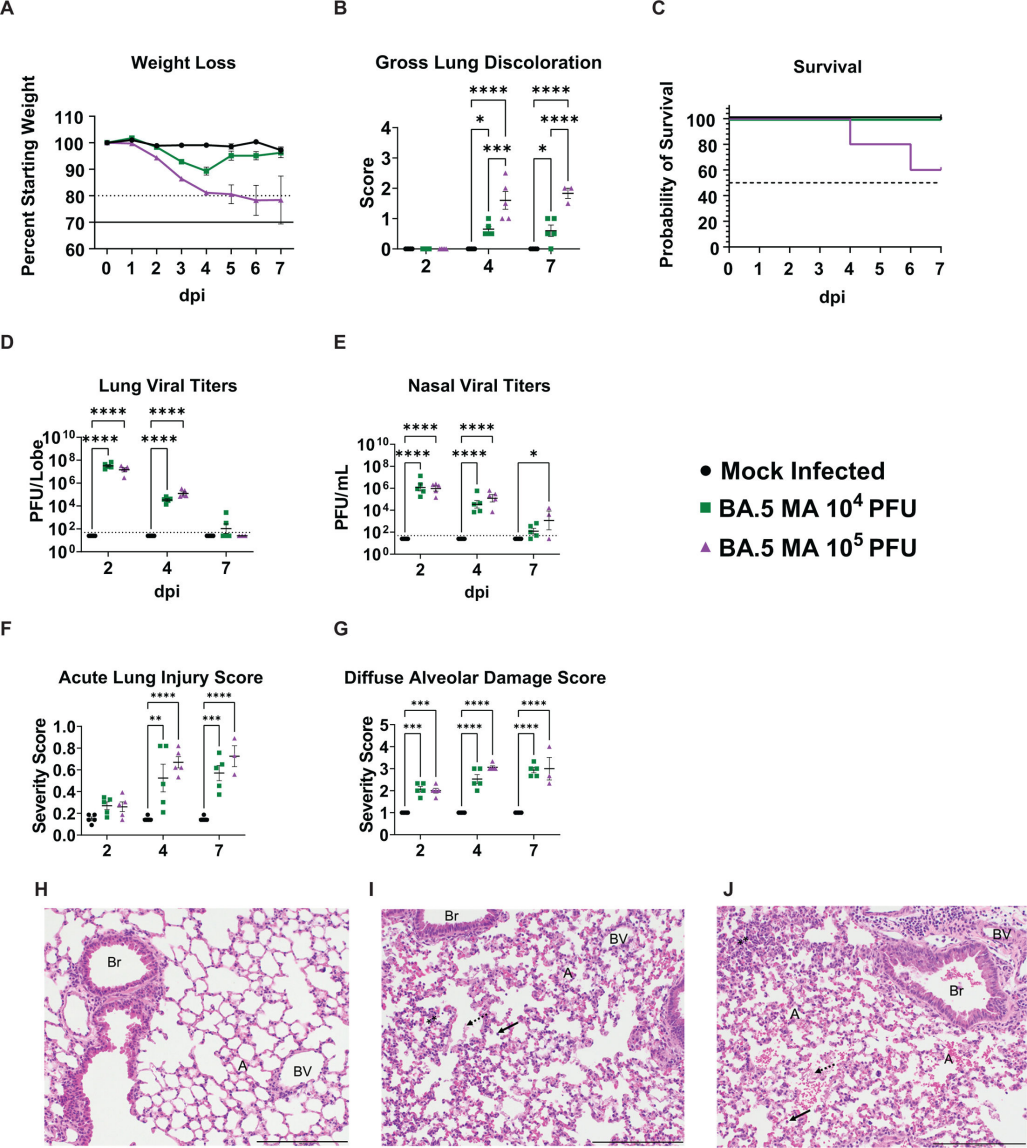
Dose-dependent pathogenicity in young mice following BA.5 MA challenge. Pathogenicity of the BA.5 MA virus was assessed in 14- to 16-week-old female BALB/c mice to determine their applicability as a model system for pathogenicity studies. (**A**) Weight loss was tracked daily for PBS control cohorts, as well as 10^4^ and 10^5^ PFU BA.5-MA-infected mice. Dashed line represents 20% wt loss, and solid line represents 30% wt loss, a humane euthanasia criterion. (**B**) GLD was evaluated and scored at the indicated timepoints of 2, 4, and 7 dpi. (**C**) Survival analysis of mice in the seven dpi cohorts identified only a dose of 10^5^ PFU resulted in mortality. (**D and E**) Virus titers were determined for replication-competent virus by plaque assay following homogenization of lung or nasal turbinate tissues. Dashed line represents limit of detection. (**F and G**) Lung damage was evaluated by two metrics, either Matute-Bello acute lung injury scoring in panel F or by diffuse alveolar damage scores in panel G. (H–J) Representative histopathological sections of lungs from PBS (**H**), 10^4^ PFU BA.5 MA (**I**), or 10^5^ PFU BA.5 MA-infected mice (**J**) at 4 days post-infection are shown (A, alveoli; Br, bronchiole; BV, blood vessel; stippled arrows, proteinaceous debris; ⁎⁎, hypercellular alveoli; solid arrow, presence of infiltrating cells in alveolar spaces). Scale bar represents 200 µm. Symbols in panels B and D–G denote statistically significant relationships at the respective levels: *, *P* < 0.05; **, *P* < 0.01; ***, *P* < 0.001; ****, *P* < 0.0001.

We have previously established a model of SARS-CoV-2 pathogenesis using mouse-adapted SARS-CoV-2 MA10 in female BALB/c mice (15). To evaluate BA.5 MA pathogenesis in a similar model, we infected 14- to 16-week-old adult female BALB/c mice intranasally with different doses, 10^4^ or 10^5^ plaque-forming units (PFUs), evaluating dose-dependent disease outcomes. While both cohorts experienced weight loss, the low-dose cohort only experienced a 10.7% group mean weight loss nadir at 4 days post-infection (dpi). In contrast, the high-dose cohort continued to lose weight throughout the study, eventually exceeding 20% of starting group mean weights (Fig. 2A). Gross lung discoloration (GLD) score is a semi-quantitative measure of acute lung damage associated with emerging CoV replication, indicative of edema and diffuse alveolar damage (15, 25). Like weight loss, GLD scores were significantly increased in the high-dose cohort as compared to the low-dose group (Fig. 2B). Similarly, mortality was only observed in the high-dose group (Fig. 2C). Regardless of inoculum dosage, the levels of viral replication within the lungs and nasal turbinates showed no significant difference between the two cohorts (Fig. 2D and E). Acute lung injury (Fig. 2F) and diffuse alveolar damage (Fig. 2G) were assessed using histological scoring schema that have been utilized for multiple emerging CoVs (15, 25). Both scoring metrics (Fig. 2F and G) revealed that acute lung injury was significantly elevated regardless of virus dose, which was evident in photomicrographs of representative mock-infected (phosphate buffered saline [PBS] inoculation) (Fig. 2H), 10^4^ PFU (Fig. 2I), and 10^5^ PFU (Fig. 2J) infected animals sacrificed at 4 dpi.

### Pathological features of infection in aged mice

**Fig 3:**
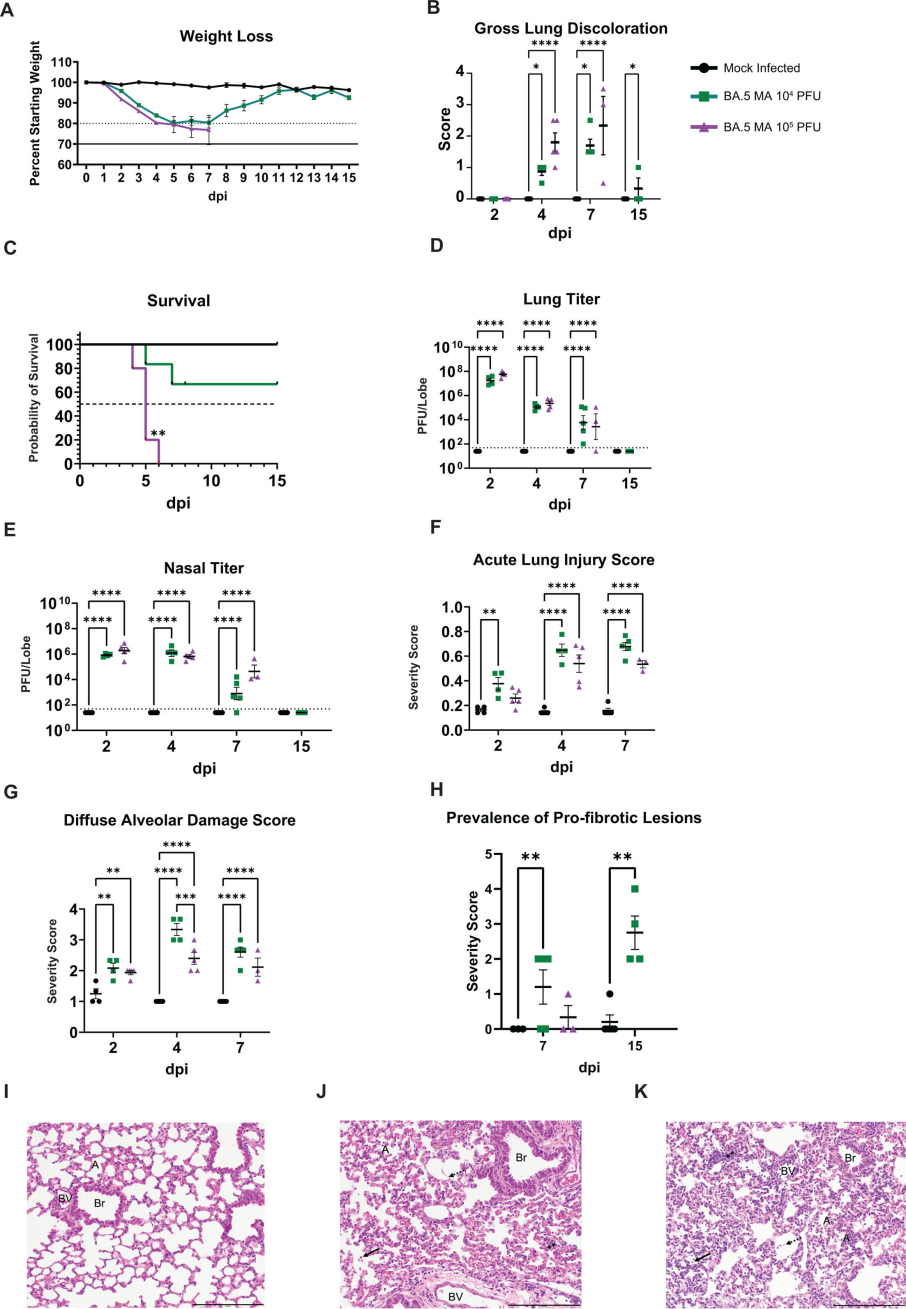
BA.5 MA induces pro-fibrotic lesions in aged mice following acute phase disease resolution. 10- to 12-month-old female BALB/c mice were used to evaluate the pathogenicity after receiving a dose of either 10^4^ or 10^5^ PFU of BA.5 MA. (**A**) Weight loss was recorded daily over the course of the study. The dashed line represents 20% wt loss, and the solid line represents 30% wt loss, a humane euthanasia criterion. (**B**) GLD was recorded at each harvest timepoint. (**C**) Mortality of a representative cohort of five mice per group that were followed until the end of the study at 15 dpi (**D and E**). Plaque assays were conducted on lung and nasal turbinate tissues to determine replication-competent virus burden. Dashed lines represent the limit of detection. (**F and G**) Histopathological examination was conducted on fixed lung sections to determine lung damage as a result of challenge. (**H**) Prevalence of pro-fibrotic lesions was scored for lungs from subjects following Picrosirius red staining (0 = none; 1 = <5% of parenchyma; 2 = 6 to 10%; 3 = 11% to 50%; 4 = 51% to 95%, 5 = >95%). (**I–K**) Representative images are shown at 4 dpi for mice inoculated with PBS, 10^4^ PFU, or 10^5^ PFU (A, alveoli; Br, bronchiole; BV, blood vessel; stippled arrows, proteinaceous debris; ⁎⁎, hypercellular alveoli; solid arrow, presence of infiltrating cells in alveolar spaces). Scale bar represents 200 µm. Symbols in panels B–H denote statistically significant relationships at the respective levels: *, *P* < 0.05; **, *P* < 0.01; ***, *P* < 0.001; ****, *P* < 0.0001.

Viral pathogenesis was then evaluated in 10- to 12-month-old mice infected with 10^4^ or 10^5^ PFU of BA.5 MA. Both high- and low-dose cohorts rapidly lost weight approaching 80% of starting weight by 4 dpi (Fig. 3A). GLD scores were elevated in the high-dose cohort as compared to the low-dose group (Fig. 3B). Although trends in body weight loss were similar up to 5 dpi, high-dose infection was uniformly lethal (Fig. 3C), while the majority of low-dose infected animals survived out to 15 dpi. As observed in the younger mice above, lung and nasal titers between the infected groups did not differ significantly, regardless of dose (Fig. 3D and E). As expected, infection was associated with increases in the histopathological measures of acute lung injury (Fig. 3F) and diffuse alveolar damage (Fig. 3G). Lung tissue sections were evaluated for fibrotic lesions by Picrosirius red staining (26). Like acute lung injury scores, the frequency of profibrotic lesions increased with infection (Fig. 3H). At later times post-infection (7, 15 dpi), we evaluated tissue sections for the prevalence of pro-fibrotic regions (Fig. 3H). Like before, only the low-dose group had elevated fibrosis scores over mock-infected animals, and importantly, no high-dose animals survived to 15 dpi and thus could not be included in this analysis. Counterintuitively, the low-dose group had elevated histological scores (Fig. 3F through H) as compared to the high-dose group, but this was likely driven by survivor bias since the few high-dose infected animals that survived to later times post-infection likely had diminished disease severity compared to those that perished at earlier times. Histologic manifestations of acute lung injury, including accumulation of proteinaceous debris in the airways, inflammatory cell infiltration, and alveolar septal thickening are shown in Fig. 3I through K.

### Monoclonal antibody treatment abrogates BA.5 MA-inflicted disease

**Fig 4:**
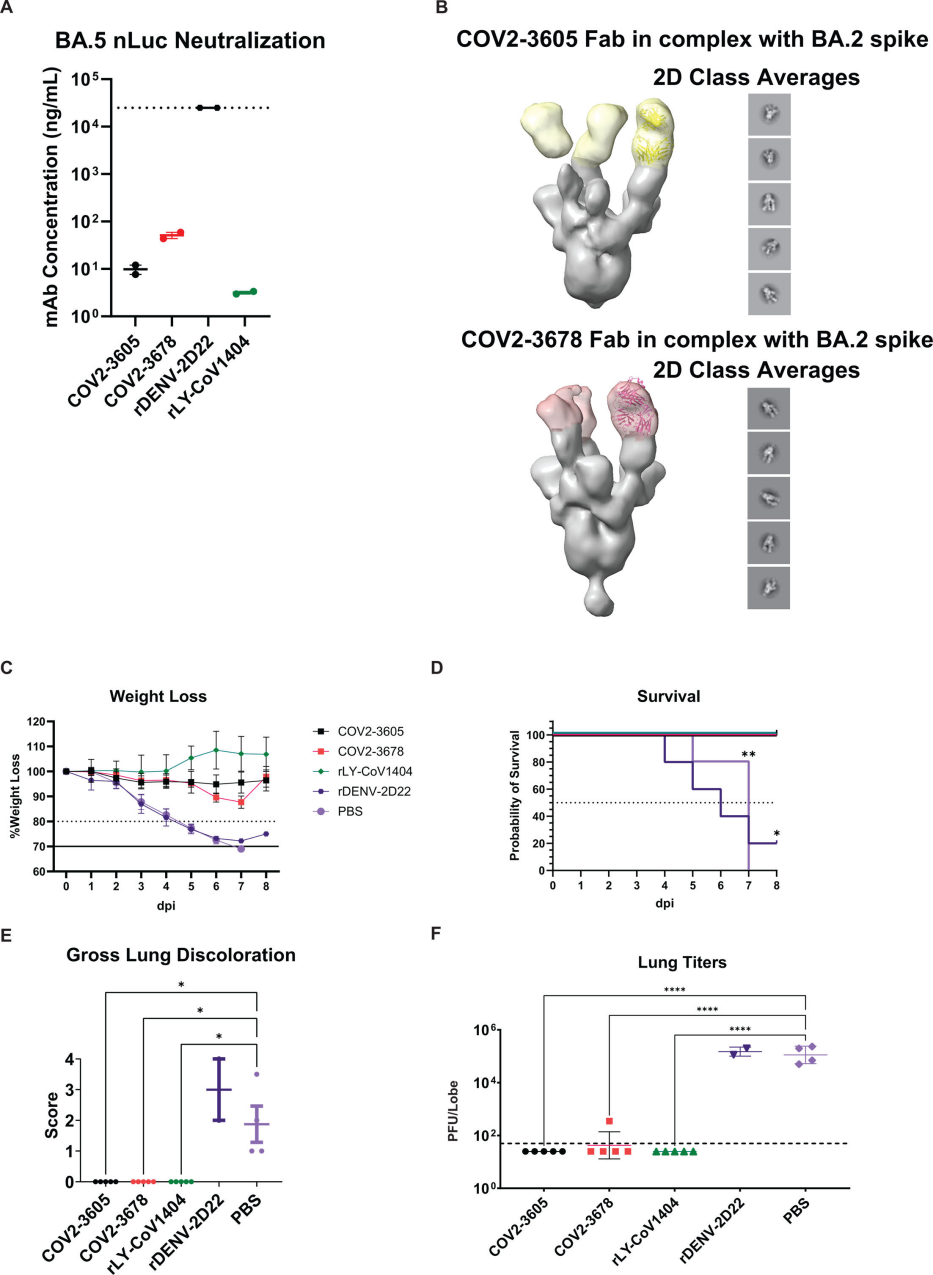
Candidate monoclonal antibodies abrogate disease pathology. 10- to 12-month-old female mice were dosed prophylactically with 200 µg of mAb or PBS intraperitoneally 12 hours prior to challenge. (**A**) Neutralization potency of mAbs was assessed against BA.5 nLuc. (**B**) Negative-stain EM of COV2-3605 and COV2-3678 Fabs in complex with BA.2 S protein shows these mAbs recognize the RBD in the up conformation and likely bind the Class I antigenic site. (**C**) Weight loss was recorded daily over the course of the study. The dashed line represents 20% wt loss, while the solid line represents 30% wt loss, a humane euthanasia criterion. (**D**) Cohort survival was evaluated for a subset of mice (*n* = 5) that was followed for the entire duration of the study. (**E**) GLD was observed at the time of mouse sacrifice. (**F**) Lung titers were determined via plaque assay to assess the quantity of replication-competent virus in lung tissue taken from mice sacrificed at 4 dpi. The dashed line represents the limit of detection. Symbols in panels D–F denote statistically significant relationships at the respective levels: *, *P* < 0.05; **, *P* < 0.01; ****, *P* < 0.0001.

From a selected panel of representative SARS-CoV-2 reactive human monoclonal antibodies (mAbs), we identified two that showed robust neutralization titers against BA.5 nLuc (Fig. 4A). Two mAbs (COV2-3605 and COV2-3678) were compared against a recombinant version of a neutralizing SARS-CoV-2 monoclonal antibody (rLY-CoV1404) that served as a positive control, and an isotype-matched recombinant version of a human mAb antibody recognizing the unrelated dengue virus envelope protein (rDENV-2D22) that served as a negative control. In addition, a mock treatment group received an equivalent volume of PBS. In a neutralization assay against BA.5 nLuc, all three SARS-CoV-2 antibodies potently neutralized the virus, with IC_50_ titers of 9.7 ng/mL for COV2-3605, 51.7 ng/mL for COV2-3678, and 3.2 ng/mL for rLY-CoV1404 (Fig. 4A). Both, COV2-3605 and COV2-3678 are encoded by the human antibody variable gene segment *IGHV3-53*. Many *IGHV3-53*- and *IGHV3-66*-encoded mAbs constitute a public clonotype, and this class of antibodies has been recurrently isolated from human subjects following SARS-CoV-2 infection or vaccination (27–30). Negative-stain electron microscopy (EM) revealed COV2-3605 Fab and COV2-3678 Fab bound to RBDs in the open conformation of the spike, consistent with the fact that members of this public clonotype target the semi-cryptic class I antigenic site (Fig. 4B) (31). Negative-stain EM data collection statistics are provided in Table S1.

To assess the prophylactic efficacy of mAbs, 10- to 12-month-old female BALB/c mice were treated with antibodies via intraperitoneal injection of 200 µg of mAb, and 12 hours later, mice were inoculated with a lethal dose of 10^5^ PFU of BA.5 MA. Mice treated with COV2-3605 or rLY-CoV1404 showed minimal or no weight loss. In contrast, ~10% transient weight loss was observed at 5 and 6 dpi for the COV2-3678-treated cohort (Fig. 4C). Importantly, negative control group mice (i.e., PBS or isotype-matched control mAb rDENV-2D22) showed rapid weight loss through 7 dpi, with all animals succumbing to infection or reaching humane endpoints for euthanasia by 7–8 dpi (Fig. 4D). Similarly, only negative control group animals had elevated GLD scores (Fig. 4E). Minimal breakthrough infection was noted in the COV2-3678-treated mice, with one of five mice exhibiting low replicating virus at 4 dpi (Fig. 4F). In contrast, no live virus was detected in the COV2-3605 and rLY-CoV1404 mAb-treated cohorts. Viral titers in mAb-treated cohorts were significantly reduced compared to PBS-treated cohorts, which had titers approaching 10^5^ PFU/lobe (*P* < 0.0001).

### Modeling Omicron-associated post-acute lung pathology with BA.2 and BA.5 MA

**Fig 5:**
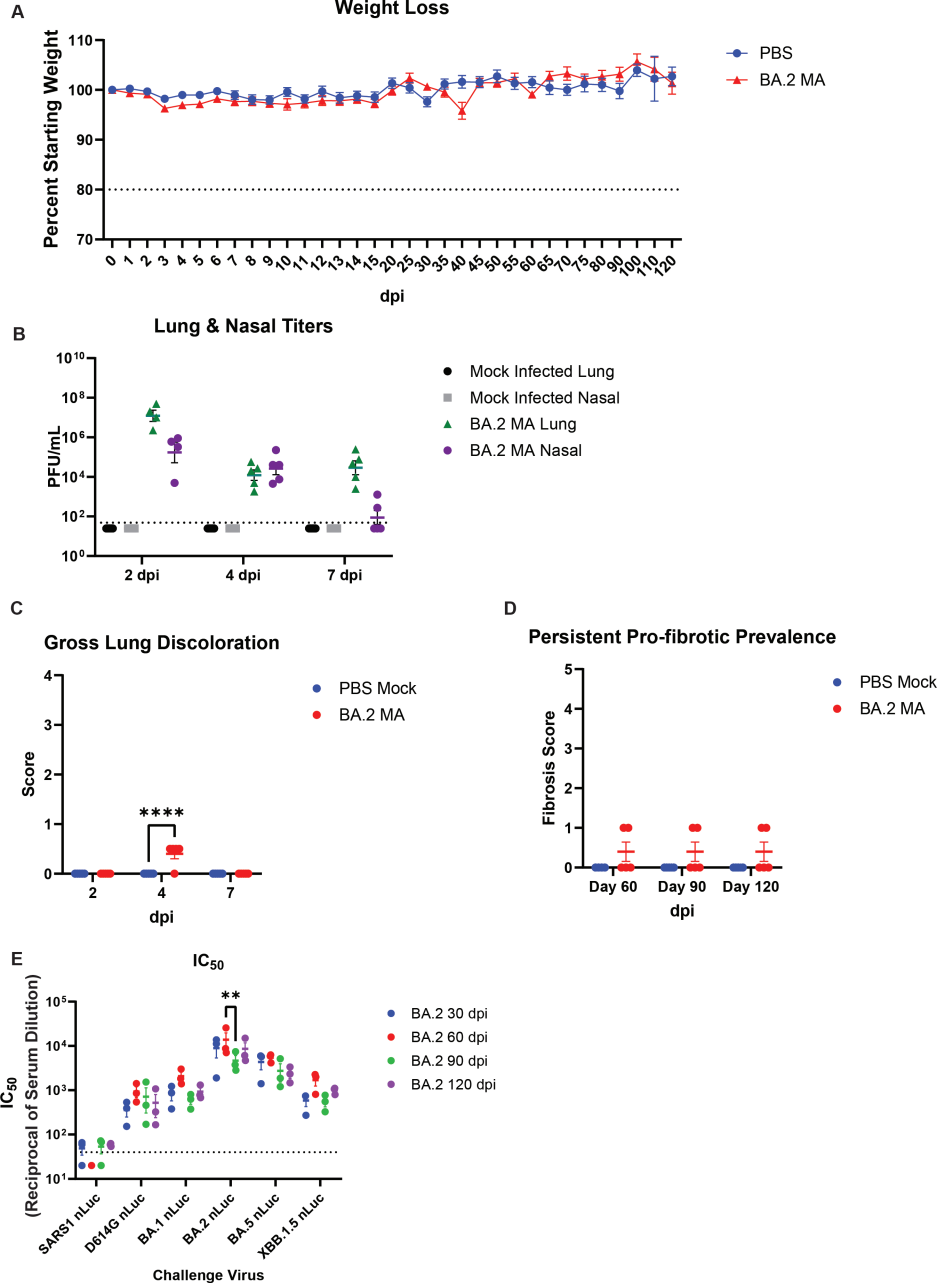
Acute and chronic disease trajectory of BA.2 MA-infected mice. 10- to 12-month-old female mice were inoculated with 10^5^ PFU of BA.2 MA. Following challenge cohorts of mice were observed out to 120 dpi with planned sacrifices at 2, 4, 7, 15, 30, 60, 90, and 120 dpi. (**A**) Mouse weights were recorded daily through the first 15 days of the experiment, after which monitoring was conducted every 5 days until day 80, and subsequently every 10 days until the terminal harvest. (**B**) Lung and nasal turbinate titers were assessed in cohort members at 2, 4, and 7 dpi and compared to mock-infected controls. The dashed line indicates the limit of detection. (**C**) GLD scores were evaluated at the acute infection timepoints. (**D**) Fibrotic scores of the lung parenchyma were assessed on samples collected at 60, 90, and 120 dpi. (**E**) Live-virus neutralization assays were performed on serum collected from mice at the indicated collection timepoints. Sera were assayed against SARS-CoV and SARS-CoV-2 viruses expressing the D614G S protein or Omicron BA.1, BA.2, BA.5, and XBB.1.5 S proteins. Symbols denote statistically significant relationships at the respective levels: **, *P* < 0.01 and ****, *P* < 0.0001.

**Fig 6:**
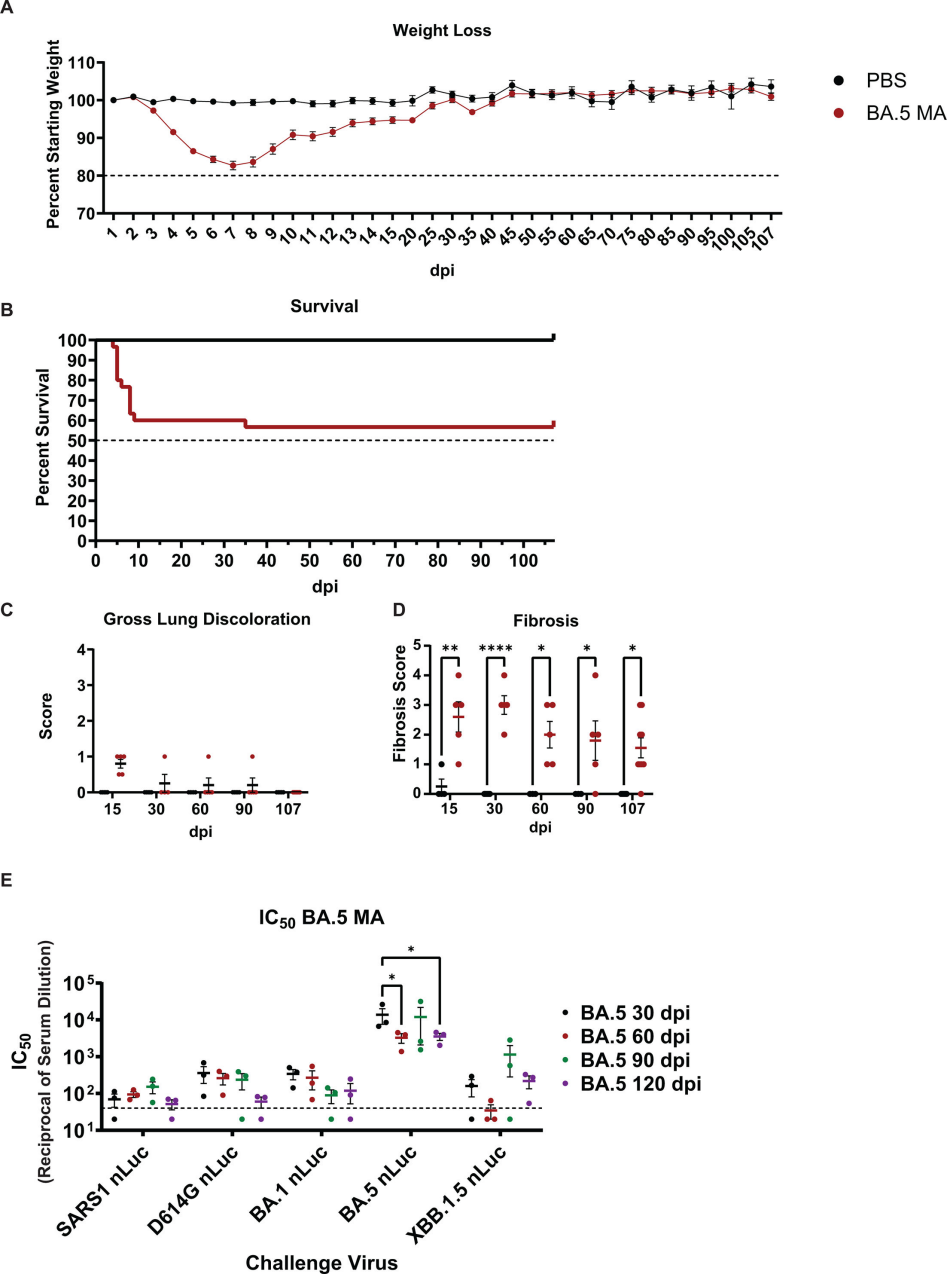
BA.5 MA induces chronic lung disease phenotypes and induces persistent homotypic neutralizing antibodies. 10- to 12-month-old female mice were inoculated with 10^4^ PFU of BA.5 MA and followed for 107 days. (**A**) Weight loss was tracked daily over the acute phase of the infection (15 days), after which it was recorded every 5 days. (**B**) Survival was recorded for mice from the 107 dpi cohort. (**C**) Lung discoloration was evaluated at the indicated timepoints. (**D**) Histopathological examination was performed on Picrosirius red-stained lung sections to determine the presence of fibrotic lesions in the lung parenchyma. (**E**) Live-virus neutralization assays were performed on blood serum collected from mice at the indicated collection timepoints. Sera were assayed against SARS-CoV or SARS-CoV-2 viruses expressing the D614G spike or Omicron BA.1, BA.5, and XBB.1.5 S proteins. Dashed line represents limit of detection. Symbols denote statistically significant relationships at the respective levels: *, *P* < 0.05; **, *P* < 0.01; ****, *P* < 0.0001.

We have previously developed a model of PASC-like lung pathology using a mouse-adapted ancestral pandemic strain SARS-CoV-2 MA10 in aged mice (26). Omicron-related infections are reported to cause less frequent PASC disease in humans as compared to ancestral VOCs (24). To understand the potential role of spike protein variation in this disease process, we generated a mouse-adapted recombinant BA.2 Omicron spike virus (BA.2 MA) that is different in 4 amino acids in the spike gene from the BA.5 MA spike-containing virus. To understand BA.2 MA PASC potential, we infected cohorts of 10- to 12-month-old female BALB/c mice with PBS (mock) or 10^5^ PFU of BA.2 MA and followed animals through 120 dpi (BA.2 MA) to evaluate virologic and pathologic outcomes. Unlike with the BA.5 MA infection noted above, BA.2 MA-infected mice exhibited minimal weight loss (Fig. 5A), despite viral replication in both the lung and nasal turbinates achieving comparable levels to those seen in the BA.5 MA-infected mice described above, demonstrating the role of spike protein variation on disease progression and severity (Fig. 5B). Congruent with body weight loss, gross pathology (Fig. 5C), and histologic measures of lung fibrosis (Fig. 5D) were largely absent and non-remarkable post-BA.2 MA infections. We next measured the magnitude and durability of the neutralizing antibody response in BA.2 MA challenged mice using antigenically homologous (BA.2 nLuc) or heterologous (SARS-CoV-1 nLuc, SARS-CoV-2 D614G nLuc, SARS-CoV-2 BA.1 nLuc, SARS-CoV-2 BA.5 nLuc, and SARS-CoV-2 XBB.1.5 nLuc) Sarbecoviruses (20). As expected, the homotypic neutralization response against BA.2 was the most robust (~10,000 IC_50_) (Fig. 5E). The magnitude of the heterotypic responses varied by genetic proximity to BA.2, where responses were 10-fold lower to ancestral pandemic strains (D614G) and were also reduced against more future emerging Omicron variants (e.g., BA.5, XBB1.5) (Fig. 5E). To understand the long-term consequences of BA.5 MA infection, we infected similarly aged mice noted above with PBS or 10-fold less virus (10^4^ PFU) to ensure disease with the majority of animals surviving. Congruent with Fig. 3, BA.5-infected animals lost an average of 17.4% ± 7.6% by 7 dpi but recovered by 30 dpi (Fig. 6A) with approximately 60% survival (Fig. 6B). Gross pathology GLD scores, which are most evident during the acute phase of infection (Fig. 3B), were low but measurable at 15 dpi and waned over time (Fig. 6C). Neither replication-competent virus via plaque assay nor viral RNA via quantitative reverse transcription-PCR was detected in lung tissue, and viral nucleocapsid antigen was not detected in lung, liver, kidney, or spleen tissue sections at any times assessed (data not shown). Lung fibrosis was evident by Picrosirius red staining with mean scores averaging between ~2 and 3 over time, indicating fibrotic lesions involving 6% to 50% of the lung parenchyma (Fig. 6D). By day 107, about 20% of the BA.5 MA virus-infected mice had resolved the most prominent long COVID lesions. Unlike mock-infected animals, BA.5 infection was associated with subpleural chronic alveolitis with scattered tertiary lymphoid structures and areas of PSR-stained interstitial fibrosis in the alveolar parenchyma at both 30 and 107 dpi (Fig. S2), thus confirming an association between chronic inflammation and fibrosis. Like BA.2 infection, homotypic neutralizing antibody responses were most robust at 30 dpi and waned through 107 dpi (Fig. 6E). As before, BA.5 serum poorly neutralized ancestral and more contemporary SARS-CoV-2 VOC and zoonotic strains. Overall, the magnitude of the heterotypic responses was driven by genetic proximity to the homotypic antigen like BA.2 above and demonstrated that Omicron spike variation can have a profound impact on acute and post-acute pathogenesis in our mouse model.

## DISCUSSION

SARS-CoV-2, the etiological agent responsible for the COVID-19 pandemic, has caused significant global morbidity and mortality, with excess mortality estimates approaching 20–25 million or more (32). In most populations, Omicron acute disease severity is reduced compared to ancestral VOCs but nevertheless continues to cause a significant disease burden with an estimated 32,000 to 50,000 deaths from October 2024 through June 2025 primarily concentrated among the elderly and certain immunocompromised populations (33–35). Both ancestral and contemporary SARS-CoV-2 VOC infections can cause chronic multiorgan system-level disease phenotypes, which have been termed long COVID or PASC. Long COVID disease symptoms can persist for months after resolution of acute infection (36, 37). Although long COVID symptoms are more likely to occur after primary infection and the overall rates of long COVID diagnoses are decreasing over time, millions of people have been impacted by long COVID, and there remains a risk of post-acute sequelae associated with reinfection (36, 38, 39). While some human studies suggest that persistence of viral RNA is a principal driver of PASC, the vast minority of chronic cases have little if any detection of persistent viral RNA, and a 15-day antiviral treatment with Paxlovid did not improve outcomes suggesting that at least persistent replication was not playing a major role in PASC (40, 41). Our data, and those of others, suggest that aberrant epithelial-immune cell interactions and/or lung repair defects drive chronic post-acute lung disease (42, 43). Altogether, these data show that important questions remain regarding the pathogenic mechanisms of long COVID and whether pre-existing long COVID conditions impact acute/chronic disease potential for future SARS-CoV-2 VOC or even other respiratory virus infections. Our ancestral and contemporary SARS-CoV-2 acute and chronic disease models presented herein provide a platform to systematically address these issues and assess the impact of medical countermeasures in models with more contemporary strains.

BA.5 MA infection in mice causes severe acute infection with histologic manifestations of acute lung injury (e.g., diffuse alveolar damage) reminiscent of infection with SARS-CoV-2 MA10, a mouse-adapted virus based on the original pandemic strain. Here, we find that BA.5 and BA.2 spike glycoprotein genes attenuate MA10 pathogenesis *in vivo* in aged mice, which is uniformly lethal at 10^4^ or greater dose in aged animals (15). We show an age-related exacerbation of pathogenesis like that observed in humans. Nevertheless, in humans, females are more likely to develop long COVID, and we aimed to generate data sets directly comparable to those from SARS-CoV-2 MA10-infected female mice (26, 44). Importantly, we demonstrate here that lower doses of BA.5 MA result in post-acute pathogenesis in the lung as evidenced by organizing pneumonia with tertiary lymphoid structures and fibrotic lesions through 107 dpi. Thus, this model could be leveraged to understand the impact of post-acute sequelae on subsequent viral infections, vaccine efficacy, and medical countermeasures to treat acute and chronic pathogenesis (17). In contrast, the BA.2 spike fully attenuated acute and chronic disease phenotypes in aged mice, despite replicating to similar titers at early times post-infection. The discordance among BA.2 and BA.5 MA outcomes in our model demonstrates the potential for S variation to impact acute and chronic pathogenesis. Our spike recombinant viruses were constructed within an isogenic SARS-CoV-2 background, thus genetically are only different in S at four positions (Δ69-70, L452R, F486V, and the wild-type Q493 in BA.5) all of which could be potential drivers of disease (45–48). Three of these mutations fall within the RBM of S. Previous studies have demonstrated that the L452R mutation in RBD enhanced ACE2 binding, fusogenicity, and infectivity, suggesting this mutation could be responsible for driving the differences in pathogenicity amongst BA.2 and BA.5 MA (49, 50). Notably, our group has previously demonstrated that a single amino acid polymorphism in S protein at position F486 can drive widely disparate disease outcomes in mice infected with XBB.1 MA versus XBB.1.5 MA (20). Despite BA.2 and BA.5 MA achieving similar titers in mice at 2 dpi, S variation could impact the kinetics of replication prior to 2 dpi and/or differences in epithelial cell tropism leading to the disparate outcomes in our data. Regardless of the mechanism, our data indicate that changes in ACE2 binding affinity could drive differential pathogenesis further implicating S protein as a major driver of disease.

COVID-19 humoral immunity acquired from natural infection is highly variable in magnitude and durability compared to vaccine-elicited immunity. In general, more severe infections oftentimes elicit higher and more durable neutralizing responses as compared to mild infections in humans (51–53). While the pathogeneses of BA.2 and BA.5 MA differed in our model, the levels of viral replication were similar at early times, and as such, the magnitude of the homotypic neutralizing responses was similar. However, neutralization breadth was limited, especially against heterotypic ancestral strains (e.g., SARS-CoV, SARS-CoV-2 D614G, and Omicron BA.1) and future, more distantly evolved Omicron-related VOC strains (i.e., XBB.1.5). Nevertheless, detailed study of the antibody repertoire after natural infection or vaccination provides an opportunity for the discovery of novel therapeutic monoclonal antibodies which have demonstrated clinical utility in treating COVID-19 (54, 55). Unfortunately, the emergence of the Omicron lineage was associated with a significant decline in Food and Drug Administration-approved mAb performance due to the accrual of mutations in S and especially those in the RBD and RBM (56–58). To understand the breadth of efficacy and mechanism of action of two mAbs (COV2-3605 and COV2-3678) isolated from an individual following a BA.1 breakthrough infection, we evaluated these mAbs in our BA.5 models. These mAbs target the RBM of the SARS-CoV-2 RBD and are encoded by antibody variable gene *IGHV3-53*, making them members of a previously described public clonotype using *IGHV3-53/IGHV3-66* genes. COV2-3605 and COV2-3678 potently neutralized BA.5 *in vitro* and *in vivo*, demonstrating that continued surveillance of patients with breakthrough infections can facilitate the identification of novel monoclonal antibodies with great breadth and efficacy. The use of *in vivo* models in this context is of importance as multiple factors including biodistribution, route of administration, and therapeutic windows can impact the efficacy of mAbs and their neutralizing competency compared to *in vitro* data. Importantly, the class I antigenic site targeted by members of this public clonotype continues to be a major target of human immunity and has accumulated further antigenic substitutions (59). However, some mAbs with this gene usage have been described that show extraordinary breadth of reactivity and resilience to escape (60).

In summary, we show that the Omicron spike can differentially elicit acute and chronic pathogenesis in BALB/c mice, including progression to long-COVID-like pulmonary disease. Disease severity is tunable based on the nature of the S protein and animal age. These findings could reflect the impact of initial viral infection inoculum on immediate response to the insult, perhaps suggesting that cytokine storm phenomena may be driving enhanced disease profiles at higher doses. Most critically, the model enables study of acute and chronic disease mechanisms in pulmonary and extra-pulmonary compartments and provides a platform for the development of medical countermeasures that target both acute and chronic disease manifestations. We show that homotypic immunity dominated the primary Omicron humoral response and that cross-protection prominently waned against ancestral pandemic strains. Since immunity to SARS-CoV-2 S has limited durability and S evolution is likely to continue to retain endemicity, our global immunity in decades to come is unlikely to effectively combat zoonoses from ancestral SARS-like viruses which circulate among reservoir species, providing opportunities for future emergence events (Fig. S3) (61–69). A weakness of this study is the focus on disease in female mice, as studies with MA10 have revealed more significant virulent acute and chronic disease phenotypes in the lungs of males (70). The study of acute and chronic CoV disease mechanisms as well as vaccine and countermeasure development remains essential for pandemic preparedness, providing a rationale for the continuation of CoV model development like those described herein.

## MATERIALS AND METHODS

### Virus and cells

Using reverse genetics, we recovered the BA.5 and BA.2 wild-type spike gene (S) sequence in the background of previously described mouse-adapted mutations (BA.5 MA and BA.2 MA) (GenBank under accession numbers PV800150 and PV800152) (20, 21). A second set of viruses encoding the BA.5 or BA.2 S protein sequence that expressed nanoluciferase gene in place of ORF7a as previously described (BA.5 nLuc and BA.2 nLuc) (GenBank under accession numbers PV800151 and PV800153) (20, 21). Infectious virus recovery was performed as previously described (17, 20). Viral growth curve analysis was performed by infecting Vero E6 cells at an MOI of 0.001.

For recombinant protein expression, Expi293F cells (Thermo Fisher Scientific; cat # A1435101) were maintained at 37°C in 8% CO_2_ in Expi293F Expression Medium (Thermo Fisher Scientific; catalog number A1435102), while ExpiCHO cells were maintained at 37°C in 8% CO_2_ in ExpiCHO Expression Medium (Thermo Fisher Scientific, cat # A2910001).

### Mice and *in vivo* infections

Female 14- to 16-week-old or 10- to 12-month-old BALB/c mice were obtained from Envigo (Inotiv) (strain 047). Mice were inoculated intranasally under ketamine/xylazine anesthesia with either 1 × 10^4^ or 1 × 10^5^ PFU BA.5 MA, or 1 × 10^5^ PFU BA.2 MA in 50 µL PBS as indicated, as previously described (15, 17, 20).

### mAb and antigen production and purification

cDNAs encoding mAbs of interest were synthesized (Twist Bioscience) and cloned into an IgG1 monocistronic expression vector (designated as pTwistmCis_G1) and used for production in mammalian cell culture. This vector contains an enhanced 2A sequence and GSG linker that allows for the simultaneous expression of mAb heavy and light chain genes from a single construct upon transfection. For antibody production, we performed transfection of ExpiCHO cell cultures using the Gibco ExpiCHO Expression System as described by the vendor (71). IgG molecules were purified from culture supernatants using HiTrap MabSelect SuRe (Cytiva) columns on a 24-column parallel protein chromatography system (Protein BioSolutions).

To express SARS-CoV-2 S proteins for ELISA binding and EM studies, we introduced the mutations of the BA.2 variant into the context of a previously described stabilized S protein construct (VFLIP) (72). In addition to a C-terminal T4 fibritin foldon domain, an 8 × His tag, and a TwinStrep tag, this construct contains an inter-protomer disulfide bond, a shorter glycine-serine-rich linker between the S1 and S2 domains, and five proline substitutions relative to the native SARS-CoV-2 S sequence. Plasmid encoding the BA.2_VFLIP antigen was transiently transfected into Expi293F cells, and culture supernatants were collected 4 to 5 days following transfection. After clarification by centrifugation and the addition of BioLock (IBA LifeSciences), antigen was purified using affinity chromatography with StrepTrap XT columns.

### EM sample preparation

EM imaging was performed with COV2-BA2 spike protein in complex with either COV2-3605 or COV2-3678. A recombinant form of the COV2-BA.2 spike was expressed and purified by affinity. Fabs were generated from purified, recombinantly expressed mAbs via enzymatic digestion using a FabALACTICA kit (Genovis, cat # A2-AFK-005). Antigen-Fab complexes were generated by incubating BA.2 S_VFLIP antigen with COV2-3605 Fab or COV2-3678 Fab in a 1:4 (antigen:Fab) molar ratio for 2 hours at room temperature.

### Negative-stain grid preparation, imaging, and processing

Three microliters of the complex sample at ~10 µg/mL was applied to a glow-discharged grid with continuous carbon film on 400 square mesh copper EM grids (Electron Microscopy Sciences). Grids were stained with 2% uranyl formate (73)⁠. Images were recorded on a Gatan US4000 4 k ´ 4 k CCD camera using an FEI TF20 (TFS) transmission electron microscope operated at 200 keV and controlled with SerialEM (74)⁠. All images were taken at 50,000 magnification with a pixel size of 2.18 Å/pixel in low-dose mode at a defocus of 1.5 to 1.8 µm. The total dose for the micrographs was ~33 e/Å2. Image processing was performed using the cryoSPARC software package (75)⁠. Images were imported, contrast transfer function estimated, and particles were picked automatically. The particles were extracted with a box size of 256 pix and binned to 128 pix (4.36 Å/pixel), and multiple rounds of 2D class averages were performed to achieve clean data sets. The final data set was used to generate an initial 3D volume, and the volume was refined for a final map at the resolution of ~18 Å. Fab Model docking to the EM map was done in Chimera. PDB: 12E8 was used for the Fab. ChimeraX⁠ software was used to make all the figures (76). Data collection statistics are provided in Table S1.

### Assessment of mAbs *in vivo*

Antibody studies were conducted with mice treated prophylactically (12 hours prior to infection) with 200 µg of the indicated mAbs or isotype-matched IgG controls. Virus-challenged mice were inoculated with BA.5 MA at 1 × 10^5^ PFU intranasally. A mock-infected control cohort received an equivalent volume of phosphate-buffered saline intranasally.

### Nanoluciferase-based neutralization assays

Moderate-throughput nanoluciferase assays in a 96-well format were conducted as previously described (20).

### Histopathology and antigen staining

Following harvest, mouse lungs were fixed for ≥7 days in 10% phosphate-buffered formalin at 4°C and prepared for histological examination and scoring as previously described (17, 20).

### Statistical analysis

All statistical analyses were performed using GraphPad Prism 10. Statistical significance was determined by two-way analysis of variance (ANOVA) with Tukey’s multiple comparison test for weight loss, Kruskal-Wallis non-parametric test with Dunn’s correction for lung discoloration, and one-way ANOVA with Tukey’s multiple comparison test for tissue titers. Symbols denote statistically significant relationships at the respective levels: *, *P* < 0.05; **, *P* < 0.01; ***, *P* < 0.001; ****, *P* < 0.0001. All cohorts started with five mice per harvest timepoint at the time of infection. Error bars represent standard error of the mean.

## Data Availability

The genomes of virus constructs used in this study are available in GenBank under accession numbers PV800150, PV800151, PV800152, and PV800153. The data sets generated in and/or analyzed in the current study are available from the corresponding authors on request.

## SUPPLEMENTAL MATERIAL

Fig. S1 jvi.01406-25-s0001.pdf Genome schematics of infectious clone viruses.

Fig. S2 jvi.01406-25-s0002.pdf Histological examination of chronically fibrotic lungs.

Fig. S3 jvi.01406-25-s0003.pdf Immunity induced by natural infection or vaccine may protect adults from early SARS-CoV-2 VOCs but not children who were exposed to later VOCs and variant vaccines.

Table S1 jvi.01406-25-s0004.pdf Electron microscopy capture statistics.

ASM does not own the copyrights to Supplemental Material that may be linked to, or accessed through, an article. The authors have granted ASM a non-exclusive, world-wide license to publish the Supplemental Material files. Please contact the corresponding author directly for reuse.

## References

[1] Kim D, Lee JY, Yang JS, Kim JW, Kim VN, Chang H. 2020. The architecture of SARS-CoV-2 transcriptome. Cell 181:914–921. doi:10.1016/j.cell.2020.04.011 32330414 PMC7179501

[2] Jungreis I, Sealfon R, Kellis M. 2021. SARS-CoV-2 gene content and COVID-19 mutation impact by comparing 44 Sarbecovirus genomes. Nat Commun 12:2642. doi:10.1038/s41467-021-22905-7 33976134 PMC8113528

[3] Lan J, Ge J, Yu J, Shan S, Zhou H, Fan S, Zhang Q, Shi X, Wang Q, Zhang L, Wang X. 2020. Structure of the SARS-CoV-2 spike receptor-binding domain bound to the ACE2 receptor. Nature 581:215–220. doi:10.1038/s41586-020-2180-5 32225176

[4] Tsai KC, Lee YC, Tseng TS. 2021. Comprehensive deep mutational scanning reveals the immune-escaping hotspots of SARS-CoV-2 receptor-binding domain targeting neutralizing antibodies. Front Microbiol 12:698365. doi:10.3389/fmicb.2021.698365 34335530 PMC8319916

[5] Greaney AJ, Loes AN, Crawford KHD, Starr TN, Malone KD, Chu HY, Bloom JD. 2021. Comprehensive mapping of mutations in the SARS-CoV-2 receptor-binding domain that affect recognition by polyclonal human plasma antibodies. Cell Host Microbe 29:463–476. doi:10.1016/j.chom.2021.02.003 33592168 PMC7869748

[6] Starr TN, Greaney AJ, Addetia A, Hannon WW, Choudhary MC, Dingens AS, Li JZ, Bloom JD. 2021. Prospective mapping of viral mutations that escape antibodies used to treat COVID-19. Science 371:850–854. doi:10.1126/science.abf9302 33495308 PMC7963219

[7] Surie D, Bonnell L, Adams K, Gaglani M, Ginde AA, Douin DJ, Talbot HK, Casey JD, Mohr NM, Zepeski A, et al.. 2022. Effectiveness of monovalent mRNA vaccines against COVID-19-associated hospitalization among immunocompetent adults during BA.1/BA.2 and BA.4/BA.5 predominant periods of SARS-CoV-2 Omicron variant in the United States — IVY Network, 18 states, December 26, 2021–August 31, 2022. MMWR Morb Mortal Wkly Rep 71:1327–1334. doi:10.15585/mmwr.mm7142a3 36264830 PMC9590291

[8] Takashita E, Yamayoshi S, Simon V, van Bakel H, Sordillo EM, Pekosz A, Fukushi S, Suzuki T, Maeda K, Halfmann P, Sakai-Tagawa Y, Ito M, Watanabe S, Imai M, Hasegawa H, Kawaoka Y. 2022. Efficacy of antibodies and antiviral drugs against omicron BA.2.12.1, BA.4, and BA.5 subvariants. N Engl J Med 387:468–470. doi:10.1056/NEJMc2207519 35857646 PMC9342381

[9] Suryawanshi RK, Chen IP, Ma T, Syed AM, Brazer N, Saldhi P, Simoneau CR, Ciling A, Khalid MM, Sreekumar B, et al.. 2022. Limited cross-variant immunity from SARS-CoV-2 Omicron without vaccination. Nature 607:351–355. doi:10.1038/s41586-022-04865-0 35584773 PMC9279157

[10] Rizvi ZA, Dandotiya J, Sadhu S, Khatri R, Singh J, Singh V, Adhikari N, Sharma K, Das V, Pandey AK, Das B, Medigeshi G, Mani S, Bhatnagar S, Samal S, Pandey AK, Garg PK, Awasthi A. 2023. Omicron sub-lineage BA.5 infection results in attenuated pathology in hACE2 transgenic mice. Commun Biol 6:935. doi:10.1038/s42003-023-05263-6 37704701 PMC10499788

[11] Uraki R, Halfmann PJ, Iida S, Yamayoshi S, Furusawa Y, Kiso M, Ito M, Iwatsuki-Horimoto K, Mine S, Kuroda M, et al.. 2022. Characterization of SARS-CoV-2 Omicron BA.4 and BA.5 isolates in rodents. Nature 612:540–545. doi:10.1038/s41586-022-05482-7 36323336 PMC12927073

[12] Stewart R, Yan K, Ellis SA, Bishop CR, Dumenil T, Tang B, Nguyen W, Larcher T, Parry R, Sng JDJ, Khromykh AA, Sullivan RKP, Lor M, Meunier FA, Rawle DJ, Suhrbier A. 2023. SARS-CoV-2 omicron BA.5 and XBB variants have increased neurotropic potential over BA.1 in K18-hACE2 mice and human brain organoids. Front Microbiol 14:1320856. doi:10.3389/fmicb.2023.1320856 38075874 PMC10706942

[13] Hoffmann M, Wong L-YR, Arora P, Zhang L, Rocha C, Odle A, Nehlmeier I, Kempf A, Richter A, Halwe NJ, Schön J, Ulrich L, Hoffmann D, Beer M, Drosten C, Perlman S, Pöhlmann S. 2023. Omicron subvariant BA.5 efficiently infects lung cells. Nat Commun 14:3500. doi:10.1038/s41467-023-39147-4 37311762 PMC10262933

[14] Shuai H, Chan JF-W, Hu B, Chai Y, Yoon C, Liu H, Liu Y, Shi J, Zhu T, Hu J-C, et al.. 2023. The viral fitness and intrinsic pathogenicity of dominant SARS-CoV-2 Omicron sublineages BA.1, BA.2, and BA.5. EBioMedicine 95:104753. doi:10.1016/j.ebiom.2023.104753 37579626 PMC10448076

[15] Leist SR, Dinnon KH III, Schäfer A, Tse LV, Okuda K, Hou YJ, West A, Edwards CE, Sanders W, Fritch EJ, Gully KL, Scobey T, Brown AJ, Sheahan TP, Moorman NJ, Boucher RC, Gralinski LE, Montgomery SA, Baric RS. 2020. A mouse-adapted SARS-CoV-2 induces acute lung injury and mortality in standard laboratory mice. Cell 183:1070–1085. doi:10.1016/j.cell.2020.09.050 33031744 PMC7510428

[16] Hou YJ, Okuda K, Edwards CE, Martinez DR, Asakura T, Dinnon KH 3rd, Kato T, Lee RE, Yount BL, Mascenik TM, et al.. 2020. SARS-CoV-2 reverse genetics reveals a variable infection gradient in the respiratory tract. Cell 182:429–446. doi:10.1016/j.cell.2020.05.042 32526206 PMC7250779

[17] Dinnon KH III, Leist SR, Schäfer A, Edwards CE, Martinez DR, Montgomery SA, West A, Yount BL Jr, Hou YJ, Adams LE, Gully KL, Brown AJ, Huang E, Bryant MD, Choong IC, Glenn JS, Gralinski LE, Sheahan TP, Baric RS. 2020. A mouse-adapted model of SARS-CoV-2 to test COVID-19 countermeasures. Nature 586:560–566. doi:10.1038/s41586-020-2708-8 32854108 PMC8034761

[18] Feng Y, Yuan M, Powers JM, Hu M, Munt JE, Arunachalam PS, Leist SR, Bellusci L, Kim J, Sprouse KR, et al.. 2023. Broadly neutralizing antibodies against sarbecoviruses generated by immunization of macaques with an AS03-adjuvanted COVID-19 vaccine. Sci Transl Med 15:eadg7404. doi:10.1126/scitranslmed.adg7404 37163615 PMC11032722

[19] Martinez DR, Schäfer A, Leist SR, Li D, Gully K, Yount B, Feng JY, Bunyan E, Porter DP, Cihlar T, Montgomery SA, Haynes BF, Baric RS, Nussenzweig MC, Sheahan TP. 2021. Prevention and therapy of SARS-CoV-2 and the B.1.351 variant in mice. Cell Rep 36:109450. doi:10.1016/j.celrep.2021.109450 34289384 PMC8270748

[20] Powers JM, Leist SR, Mallory ML, Yount BL, Gully KL, Zweigart MR, Bailey AB, Sheahan TP, Harkema JR, Baric RS. 2024. Divergent pathogenetic outcomes in BALB/c mice following Omicron subvariant infection. Virus Res 341:199319. doi:10.1016/j.virusres.2024.199319 38224840 PMC10835285

[21] Parotto M, Gyöngyösi M, Howe K, Myatra SN, Ranzani O, Shankar-Hari M, Herridge MS. 2023. Post-acute sequelae of COVID-19: understanding and addressing the burden of multisystem manifestations. Lancet Respir Med 11:739–754. doi:10.1016/S2213-2600(23)00239-4 37475125

[22] Huang L, Yao Q, Gu X, Wang Q, Ren L, Wang Y, Hu P, Guo L, Liu M, Xu J, Zhang X, Qu Y, Fan Y, Li X, Li C, Yu T, Xia J, Wei M, Chen L, Li Y, Xiao F, Liu D, Wang J, Wang X, Cao B. 2021. 1-year outcomes in hospital survivors with COVID-19: a longitudinal cohort study. The Lancet 398:747–758. doi:10.1016/S0140-6736(21)01755-4 PMC8389999 34454673

[23] Mandel H, Yoo YJ, Allen AJ, Abedian S, Verzani Z, Karlson EW, Kleinman LC, Mudumbi PC, Oliveira CR, Muszynski JA, et al.. 2025. Long COVID incidence proportion in adults and children between 2020 and 2024: an electronic health record-based study from the RECOVER initiative. Clin Infect Dis 80:1247–1261. doi:10.1093/cid/ciaf046 39907495 PMC12272849

[24] National Academies of Sciences, Engineering, and Medicine. 2024. A long COVID definition: a chronic, systemic disease state with profound consequences. The National Academies Press, Washington, DC. 39110819

[25] Sheahan TP, Sims AC, Leist SR, Schäfer A, Won J, Brown AJ, Montgomery SA, Hogg A, Babusis D, Clarke MO, Spahn JE, Bauer L, Sellers S, Porter D, Feng JY, Cihlar T, Jordan R, Denison MR, Baric RS. 2020. Comparative therapeutic efficacy of remdesivir and combination lopinavir, ritonavir, and interferon beta against MERS-CoV. Nat Commun 11:222. doi:10.1038/s41467-019-13940-6 31924756 PMC6954302

[26] Dinnon KH III, Leist SR, Okuda K, Dang H, Fritch EJ, Gully KL, De la Cruz G, Evangelista MD, Asakura T, Gilmore RC, et al.. 2022. SARS-CoV-2 infection produces chronic pulmonary epithelial and immune cell dysfunction with fibrosis in mice. Sci Transl Med 14:eabo5070. doi:10.1126/scitranslmed.abo5070 35857635 PMC9273046

[27] Kim SI, Noh J, Kim S, Choi Y, Yoo DK, Lee Y, Lee H, Jung J, Kang CK, Song K-H, Choe PG, Kim HB, Kim ES, Kim N-J, Seong M-W, Park WB, Oh M-D, Kwon S, Chung J. 2021. Stereotypic neutralizing V_H_ antibodies against SARS-CoV-2 spike protein receptor binding domain in patients with COVID-19 and healthy individuals. Sci Transl Med 13:eabd6990. doi:10.1126/scitranslmed.abd6990 33397677 PMC7875332

[28] Yuan M, Liu H, Wu NC, Lee C-CD, Zhu X, Zhao F, Huang D, Yu W, Hua Y, Tien H, Rogers TF, Landais E, Sok D, Jardine JG, Burton DR, Wilson IA. 2020. Structural basis of a shared antibody response to SARS-CoV-2. Science 369:1119–1123. doi:10.1126/science.abd2321 32661058 PMC7402627

[29] Li L, Chen X, Wang Z, Li Y, Wang C, Jiang L, Zuo T. 2023. Breakthrough infection elicits hypermutated IGHV3-53/3-66 public antibodies with broad and potent neutralizing activity against SARS-CoV-2 variants including the emerging EG.5 lineages. PLoS Pathog 19:e1011856. doi:10.1371/journal.ppat.1011856 38048356 PMC10721163

[30] Tan TJC, Yuan M, Kuzelka K, Padron GC, Beal JR, Chen X, Wang Y, Rivera-Cardona J, Zhu X, Stadtmueller BM, Brooke CB, Wilson IA, Wu NC. 2021. Sequence signatures of two public antibody clonotypes that bind SARS-CoV-2 receptor binding domain. Nat Commun 12:3815. doi:10.1038/s41467-021-24123-7 34155209 PMC8217500

[31] Greaney AJ, Starr TN, Barnes CO, Weisblum Y, Schmidt F, Caskey M, Gaebler C, Cho A, Agudelo M, Finkin S, Wang Z, Poston D, Muecksch F, Hatziioannou T, Bieniasz PD, Robbiani DF, Nussenzweig MC, Bjorkman PJ, Bloom JD. 2021. Mapping mutations to the SARS-CoV-2 RBD that escape binding by different classes of antibodies. Nat Commun 12:4196. doi:10.1038/s41467-021-24435-8 34234131 PMC8263750

[32] Wang H, Paulson KR, Pease SA, Watson S, Comfort H, Zheng P, Aravkin AY, Bisignano C, Barber RM, Alam T, et al.. 2022. Estimating excess mortality due to the COVID-19 pandemic: a systematic analysis of COVID-19-related mortality, 2020–21. The Lancet 399:1513–1536. doi:10.1016/S0140-6736(21)02796-3 PMC8912932 35279232

[33] Navarrete J, Barone G, Qureshi I, Woods A, Barbre K, Meng L, Novosad S, Li Q, Soe MM, Edwards J, Wong E, Reses HE, Guthrie S, Keenan J, Lamping L, Park M, Dumbuya S, Benin AL, Bell J. 2023. SARS-CoV-2 infection and death rates among maintenance dialysis patients during Delta and early Omicron waves — United States, June 30, 2021–September 27, 2022. MMWR Morb Mortal Wkly Rep 72:871–876. doi:10.15585/mmwr.mm7232a4 37561674 PMC10415006

[34] Hedberg P, Parczewski M, Serwin K, Marchetti G, Bai F, Ole Jensen B-E, Pereira JPV, Drobniewski F, Reschreiter H, Naumovas D, Ceccherini-Silberstein F, Rubio Quintanares GH, Mwau M, Toscano C, König F, Pfeifer N, Zazzi M, Fanti I, Incardona F, Cozzi-Lepri A, Sönnerborg A, Nauclér P. 2024. In-hospital mortality during the wild-type, alpha, delta, and omicron SARS-CoV-2 waves: a multinational cohort study in the EuCARE project. Lancet Reg Health Eur 38:100855. doi:10.1016/j.lanepe.2024.100855 38476753 PMC10928271

[35] Prevention USCfDCa. 2024. Preliminary estimates of COVID-19 burden for 2024-2025. Available from: https://www.cdc.gov/covid/php/surveillance/burden-estimates.html

[36] Davis HE, McCorkell L, Vogel JM, Topol EJ. 2023. Long COVID: major findings, mechanisms and recommendations. Nat Rev Microbiol 21:133–146. doi:10.1038/s41579-022-00846-2 36639608 PMC9839201

[37] Su S, Zhao Y, Zeng N, Liu X, Zheng Y, Sun J, Zhong Y, Wu S, Ni S, Gong Y, Zhang Z, Gao N, Yuan K, Yan W, Shi L, Ravindran AV, Kosten T, Shi J, Bao Y, Lu L. 2023. Epidemiology, clinical presentation, pathophysiology, and management of long COVID: an update. Mol Psychiatry 28:4056–4069. doi:10.1038/s41380-023-02171-3 37491461

[38] Hadley E, Yoo YJ, Patel S, Zhou A, Laraway B, Wong R, Preiss A, Chew R, Davis H, Brannock MD, Chute CG, Pfaff ER, Loomba J, Haendel M, Hill E, Moffitt R, N3C and RECOVER consortia. 2024. Insights from an N3C RECOVER EHR-based cohort study characterizing SARS-CoV-2 reinfections and Long COVID. Commun Med 4:129. doi:10.1038/s43856-024-00539-2 38992084 PMC11239932

[39] Bowe B, Xie Y, Al-Aly Z. 2022. Acute and postacute sequelae associated with SARS-CoV-2 reinfection. Nat Med 28:2398–2405. doi:10.1038/s41591-022-02051-3 36357676 PMC9671810

[40] Cohen AK, Jaudon TW, Schurman EM, Kava L, Vogel JM, Haas-Godsil J, Lewis D, Crausman S, Leslie K, Bligh SC, Lizars G, Davids JD, Sran S, Peluso M, McCorkell L. 2025. Impact of extended-course oral nirmatrelvir/ritonavir in established Long COVID: a case series. Commun Med 4:261. doi:10.1038/s43856-024-00668-8 39762640 PMC11704346

[41] Geng LN, Bonilla H, Hedlin H, Jacobson KB, Tian L, Jagannathan P, Yang PC, Subramanian AK, Liang JW, Shen S, et al.. 2024. Nirmatrelvir-ritonavir and symptoms in adults with postacute sequelae of SARS-CoV-2 infection: The STOP-PASC randomized clinical trial. JAMA Intern Med 184:1024–1034. doi:10.1001/jamainternmed.2024.2007 38848477 PMC11161857

[42] Wei X, Qian W, Narasimhan H, Chan T, Liu X, Arish M, Young S, Li C, Cheon IS, Yu Q, et al.. 2025. Macrophage peroxisomes guide alveolar regeneration and limit SARS-CoV-2 tissue sequelae. Science 387:eadq2509. doi:10.1126/science.adq2509 40048515 PMC12681967

[43] Schäfer A, Leist SR, Powers JM, Baric RS. 2024. Animal models of long Covid: a hit-and-run disease. Sci Transl Med 16:eado2104. doi:10.1126/scitranslmed.ado2104 39536118 PMC12269645

[44] Sudre CH, Murray B, Varsavsky T, Graham MS, Penfold RS, Bowyer RC, Pujol JC, Klaser K, Antonelli M, Canas LS, et al.. 2021. Attributes and predictors of long COVID. Nat Med 27:626–631. doi:10.1038/s41591-021-01292-y 33692530 PMC7611399

[45] Tegally H, Moir M, Everatt J, Giovanetti M, Scheepers C, Wilkinson E, Subramoney K, Makatini Z, Moyo S, Amoako DG, et al.. 2022. Emergence of SARS-CoV-2 Omicron lineages BA.4 and BA.5 in South Africa. Nat Med 28:1785–1790. doi:10.1038/s41591-022-01911-2 35760080 PMC9499863

[46] Yajima H, Nomai T, Okumura K, Maenaka K, Phenotype Japan C, Ito J, Hashiguchi T, Sato K. 2024. Molecular and structural insights into SARS-CoV-2 evolution: from BA.2 to XBB subvariants. mBio 15:e0322023. doi:10.1128/mbio.03220-23 39283095 PMC11481514

[47] Tsueng G, Mullen JL, Alkuzweny M, Cano M, Rush B, Haag E, Lin J, Welzel DJ, Zhou X, Qian Z, Latif AA, Hufbauer E, Zeller M, Andersen KG, Wu C, Su AI, Gangavarapu K, Hughes LD. 2023. Outbreak.info Research Library: a standardized, searchable platform to discover and explore COVID-19 resources. Nat Methods 20:536–540. doi:10.1038/s41592-023-01770-w 36823331 PMC10393269

[48] Gangavarapu K, Latif AA, Mullen JL, Alkuzweny M, Hufbauer E, Tsueng G, Haag E, Zeller M, Aceves CM, Zaiets K, Cano M, Zhou X, Qian Z, Sattler R, Matteson NL, Levy JI, Lee RTC, Freitas L, Maurer-Stroh S, GISAID Core and Curation Team, Suchard MA, Wu C, Su AI, Andersen KG, Hughes LD. 2023. Outbreak.info genomic reports: scalable and dynamic surveillance of SARS-CoV-2 variants and mutations. Nat Methods 20:512–522. doi:10.1038/s41592-023-01769-3 36823332 PMC10399614

[49] Zhang Y, Zhang T, Fang Y, Liu J, Ye Q, Ding L. 2022. SARS-CoV-2 spike L452R mutation increases Omicron variant fusogenicity and infectivity as well as host glycolysis. Sig Transduct Target Ther 7:76. doi:10.1038/s41392-022-00941-z PMC8905570 35264568

[50] Motozono C, Toyoda M, Zahradnik J, Saito A, Nasser H, Tan TS, Ngare I, Kimura I, Uriu K, Kosugi Y, et al.. 2021. SARS-CoV-2 spike L452R variant evades cellular immunity and increases infectivity. Cell Host Microbe 29:1124–1136. doi:10.1016/j.chom.2021.06.006 34171266 PMC8205251

[51] Choe PG, Kang CK, Suh HJ, Jung J, Kang E, Lee SY, Song KH, Kim HB, Kim NJ, Park WB, Kim ES, Oh MD. 2020. Antibody responses to SARS-CoV-2 at 8 weeks postinfection in asymptomatic patients. Emerg Infect Dis 26:2484–2487. doi:10.3201/eid2610.202211 32579877 PMC7510710

[52] Yan LN, Liu PP, Li XG, Zhou SJ, Li H, Wang ZY, Shen F, Lu BC, Long Y, Xiao X, Wang ZD, Li D, Han HJ, Yu H, Zhou SH, Lv WL, Yu XJ. 2021. Neutralizing antibodies and cellular immune responses against SARS-CoV-2 sustained one and a half years after natural infection. Front Microbiol 12:803031. doi:10.3389/fmicb.2021.803031 35310397 PMC8928406

[53] Sancilio A, Schrock JM, Demonbreun AR, D’Aquila RT, Mustanski B, Vaught LA, Reiser NL, Velez MP, Hsieh RR, Ryan DT, Saber R, McNally EM, McDade TW. 2022. COVID-19 symptom severity predicts neutralizing antibody activity in a community-based serological study. Sci Rep 12:12269. doi:10.1038/s41598-022-15791-6 35851303 PMC9293881

[54] Yu SY, Choi M, Cheong C, Ryoo S, Huh K, Yoon YK, Choi J, Kim SB. 2023. Clinical efficacy and safety of SARS-CoV-2-neutralizing monoclonal antibody in patients with COVID-19: a living systematic review and meta-analysis. J Microbiol Immunol Infect 56:909–920. doi:10.1016/j.jmii.2023.07.009 37598054

[55] Kip KE, McCreary EK, Collins K, Minnier TE, Snyder GM, Garrard W, McKibben JC, Yealy DM, Seymour CW, Huang DT, Bariola JR, Schmidhofer M, Wadas RJ, Angus DC, Kip PL, Marroquin OC. 2023. Evolving real-world effectiveness of monoclonal antibodies for treatment of COVID-19: a cohort study. Ann Intern Med 176:496–504. doi:10.7326/M22-1286 37011399 PMC10074437

[56] Cox M, Peacock TP, Harvey WT, Hughes J, Wright DW, Consortium C-G, Willett BJ, Thomson E, Gupta RK, Peacock SJ, Robertson DL, Carabelli AM. 2023. SARS-CoV-2 variant evasion of monoclonal antibodies based on in vitro studies. Nat Rev Microbiol 21:112–124. doi:10.1038/s41579-022-00809-7 36307535 PMC9616429

[57] Focosi D, McConnell S, Casadevall A, Cappello E, Valdiserra G, Tuccori M. 2022. Monoclonal antibody therapies against SARS-CoV-2. Lancet Infect Dis 22:e311–e326. doi:10.1016/S1473-3099(22)00311-5 35803289 PMC9255948

[58] Wang Q, Guo Y, Iketani S, Nair MS, Li Z, Mohri H, Wang M, Yu J, Bowen AD, Chang JY, Shah JG, Nguyen N, Chen Z, Meyers K, Yin MT, Sobieszczyk ME, Sheng Z, Huang Y, Liu L, Ho DD. 2022. Antibody evasion by SARS-CoV-2 Omicron subvariants BA.2.12.1, BA.4 and BA.5. Nature 608:603–608. doi:10.1038/s41586-022-05053-w 35790190 PMC9385487

[59] Jian F, Wang J, Yisimayi A, Song W, Xu Y, Chen X, Niu X, Yang S, Yu Y, Wang P, Sun H, Yu L, Wang J, Wang Y, An R, Wang W, Ma M, Xiao T, Gu Q, Shao F, Wang Y, Shen Z, Jin R, Cao Y. 2025. Evolving antibody response to SARS-CoV-2 antigenic shift from XBB to JN.1. Nature 637:921–929. doi:10.1038/s41586-024-08315-x 39510125 PMC11754117

[60] Jian F, Wec AZ, Feng L, Yu Y, Wang L, Wang P, Yu L, Wang J, Hou J, Berrueta DM, et al.. 2024. Viral evolution prediction identifies broadly neutralizing antibodies to existing and prospective SARS-CoV-2 variants. Nat Microbiol 10:2003–2017. doi:10.1038/s41564-025-02030-7 PMC12313522 40494884

[61] Goraichuk IV, Arefiev V, Stegniy BT, Gerilovych AP. 2021. Zoonotic and reverse zoonotic transmissibility of SARS-CoV-2. Virus Res 302:198473. doi:10.1016/j.virusres.2021.198473 34118360 PMC8188804

[62] Markov PV, Ghafari M, Beer M, Lythgoe K, Simmonds P, Stilianakis NI, Katzourakis A. 2023. The evolution of SARS-CoV-2. Nat Rev Microbiol 21:361–379. doi:10.1038/s41579-023-00878-2 37020110

[63] Yen HL, Sit THC, Brackman CJ, Chuk SSY, Gu H, Tam KWS, Law PYT, Leung GM, Peiris M, Poon LLM. 2022. Transmission of SARS-CoV-2 delta variant (AY.127) from pet hamsters to humans, leading to onward human-to-human transmission: a case study. Lancet 399:1070–1078. doi:10.1016/S0140-6736(22)00326-9 35279259 PMC8912929

[64] McBride DS, Garushyants SK, Franks J, Magee AF, Overend SH, Huey D, Williams AM, Faith SA, Kandeil A, Trifkovic S, et al.. 2023. Accelerated evolution of SARS-CoV-2 in free-ranging white-tailed deer. Nat Commun 14:5105. doi:10.1038/s41467-023-40706-y 37640694 PMC10462754

[65] Caserta LC, Martins M, Butt SL, Hollingshead NA, Covaleda LM, Ahmed S, Everts MRR, Schuler KL, Diel DG. 2023. White-tailed deer (Odocoileus virginianus) may serve as a wildlife reservoir for nearly extinct SARS-CoV-2 variants of concern. Proc Natl Acad Sci USA 120:e2215067120. doi:10.1073/pnas.2215067120 36719912 PMC9963525

[66] Pickering B, Lung O, Maguire F, Kruczkiewicz P, Kotwa JD, Buchanan T, Gagnier M, Guthrie JL, Jardine CM, Marchand-Austin A, et al.. 2022. Divergent SARS-CoV-2 variant emerges in white-tailed deer with deer-to-human transmission. Nat Microbiol 7:2011–2024. doi:10.1038/s41564-022-01268-9 36357713 PMC9712111

[67] Ge XY, Li JL, Yang XL, Chmura AA, Zhu G, Epstein JH, Mazet JK, Hu B, Zhang W, Peng C, Zhang YJ, Luo CM, Tan B, Wang N, Zhu Y, Crameri G, Zhang SY, Wang LF, Daszak P, Shi ZL. 2013. Isolation and characterization of a bat SARS-like coronavirus that uses the ACE2 receptor. Nature 503:535–538. doi:10.1038/nature12711 24172901 PMC5389864

[68] Xiao K, Zhai J, Feng Y, Zhou N, Zhang X, Zou JJ, Li N, Guo Y, Li X, Shen X, Zhang Z, Shu F, Huang W, Li Y, Zhang Z, Chen RA, Wu YJ, Peng SM, Huang M, Xie WJ, Cai QH, Hou FH, Chen W, Xiao L, Shen Y. 2020. Isolation of SARS-CoV-2-related coronavirus from Malayan pangolins. Nature 583:286–289. doi:10.1038/s41586-020-2313-x 32380510

[69] Ou X, Xu G, Li P, Liu Y, Zan F, Liu P, Hu J, Lu X, Dong S, Zhou Y, Mu Z, Wu Z, Wang J, Jin Q, Liu P, Lu J, Wang X, Qian Z. 2023. Host susceptibility and structural and immunological insight of S proteins of two SARS-CoV-2 closely related bat coronaviruses. Cell Discov 9:78. doi:10.1038/s41421-023-00581-9 37507385 PMC10382498

[70] Davis MA, Voss K, Turnbull JB, Gustin AT, Knoll M, Muruato A, Hsiang T-Y, Dinnon Iii KH, Leist SR, Nickel K, Baric RS, Ladiges W, Akilesh S, Smith KD, Gale M Jr. 2022. A C57BL/6 mouse model of SARS-CoV-2 infection recapitulates age- and sex-based differences in human COVID-19 disease and recovery. Vaccines (Basel) 11:47. doi:10.3390/vaccines11010047 36679892 PMC9860616

[71] Chng J, Wang T, Nian R, Lau A, Hoi KM, Ho SCL, Gagnon P, Bi X, Yang Y. 2015. Cleavage efficient 2A peptides for high level monoclonal antibody expression in CHO cells. MAbs 7:403–412. doi:10.1080/19420862.2015.1008351 25621616 PMC4622431

[72] Olmedillas E, Rajamanickam RR, Avalos RD, Ana-Sosa-Batiz F, Zyla D, Zandonatti MA, Harkins SS, Shresta S, Hastie KM, Saphire EO. 2025. Structure of a SARS-CoV-2 spike S2 subunit in a pre-fusion, open conformation. Cell Rep 44:116052. doi:10.1016/j.celrep.2025.116052 40705599

[73] Ohi M, Li Y, Cheng Y, Walz T. 2004. Negative staining and image classification - powerful tools in modern electron microscopy. Biol Proced Online 6:23–34. doi:10.1251/bpo70 15103397 PMC389902

[74] Mastronarde DN. 2005. Automated electron microscope tomography using robust prediction of specimen movements. J Struct Biol 152:36–51. doi:10.1016/j.jsb.2005.07.007 16182563

[75] Punjani A, Rubinstein JL, Fleet DJ, Brubaker MA. 2017. cryoSPARC: algorithms for rapid unsupervised cryo-EM structure determination. Nat Methods 14:290–296. doi:10.1038/nmeth.4169 28165473

[76] Pettersen EF, Goddard TD, Huang CC, Meng EC, Couch GS, Croll TI, Morris JH, Ferrin TE. 2021. UCSF ChimeraX: structure visualization for researchers, educators, and developers. Protein Sci 30:70–82. doi:10.1002/pro.3943 32881101 PMC7737788

