# Supplementary material for: Mouse-adapted SARS-CoV-2 Omicron BA.5 infection induces post-acute lung fibrosis in BALB/c mice
Source: J Virol. 2025 Nov 6;99(11):e01406-25. doi: 10.1128/jvi.01406-25 (PMC12645932; doi:10.1128/jvi.01406-25)
Supplement: Fig. S1 — Genome schematics of infectious clone viruses. [file jvi.01406-25-s0001.pdf]

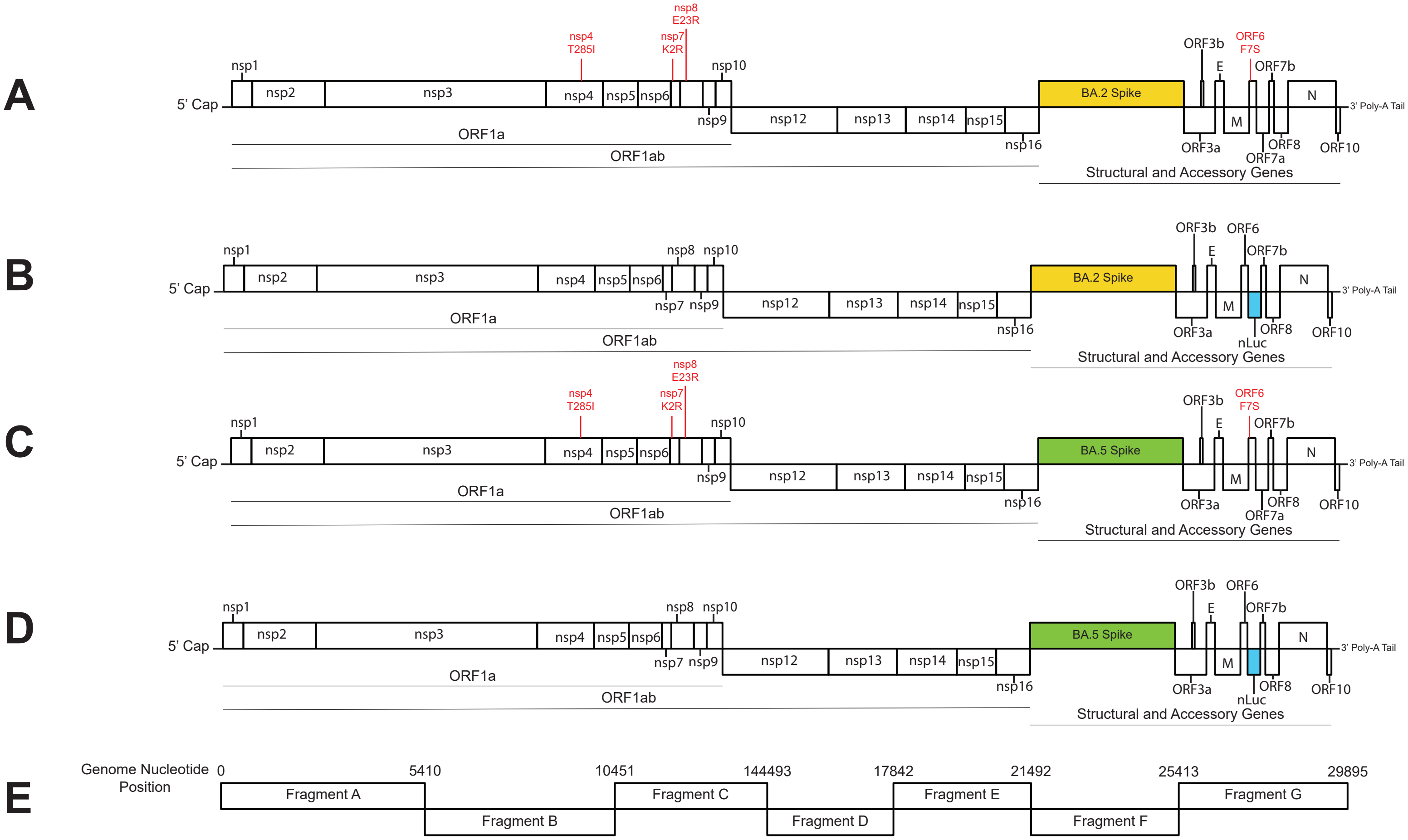

Figure S1. Genome Schematics of Infectious Clone Viruses

Genome schematics are shown for BA.2 and BA.5 virus constructs. (**A**) Genome of the BA.2 MA virus. Mouse adaptation mutations are shown in red. Spike gene is shown in yellow. (**B**) Genome of the BA.2 nLuc virus. Location of the nanoluciferase cassette is shown in teal. Spike gene is shown in yellow. (**C**) Genome of the BA.5 MA virus. Mouse adaptation mutations are shown in red. Spike gene is shown in green. (**D**) Genome of the BA.5 nLuc virus. Location of the nanoluciferase cassette is shown in teal. Spike gene is shown in green. (**E**) Fragments of the infectious clone system are shown, with the genome nucleotide positions annotated.
