# Supplementary material for: Mouse-adapted SARS-CoV-2 Omicron BA.5 infection induces post-acute lung fibrosis in BALB/c mice
Source: J Virol. 2025 Nov 6;99(11):e01406-25. doi: 10.1128/jvi.01406-25 (PMC12645932; doi:10.1128/jvi.01406-25)
Supplement: Fig. S2 — Histological examination of chronically fibrotic lungs. [file jvi.01406-25-s0002.pdf]

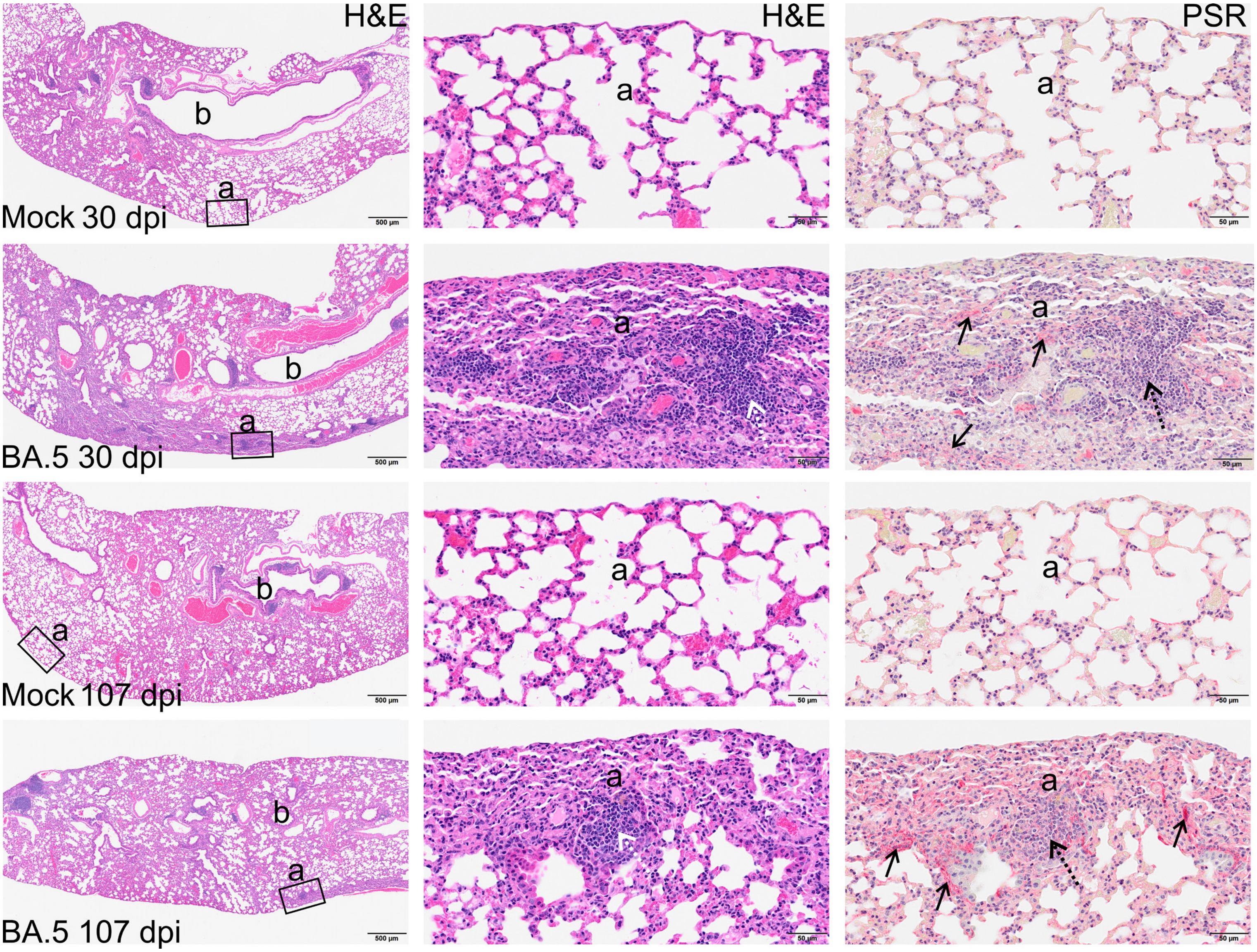

Figure S2. Histological Examination of Chronically Fibrotic Lungs

Representative images of histological samples used to score fibrotic disease as reported in **Fig. 6E.** Light photomicrographs, at low (scale bar 500 µm) or high magnifications (scale bar 50 µm), of left lung lobe tissue sections stained with hematoxylin and eosin (H&E) or Picrosirius red (PSR). Lung lobes were taken at 30- or 107-days post infection (dpi) from mock-infected mice (controls) or mice intranasally instilled with BA.5. Pulmonary histopathology was not observed in mock-infected mice. In contrast, BA.5 mice at 30 or 107 dpi had subpleural chronic alveolitis with scattered tertiary lymphoid structures (stippled arrows) and areas of PSR-stained interstitial fibrosis in the alveolar parenchyma (a). (b) bronchiolar airways; small rectangular boxes, location of the photos taken at high magnification.
