# Supplementary material for: Mouse-adapted SARS-CoV-2 Omicron BA.5 infection induces post-acute lung fibrosis in BALB/c mice
Source: J Virol. 2025 Nov 6;99(11):e01406-25. doi: 10.1128/jvi.01406-25 (PMC12645932; doi:10.1128/jvi.01406-25)
Supplement: Fig. S3 — Immunity induced by natural infection or vaccine may protect adults from early SARS-CoV-2 VOCs but not children who were exposed to later VOCs and variant vaccines. [file jvi.01406-25-s0003.pdf]

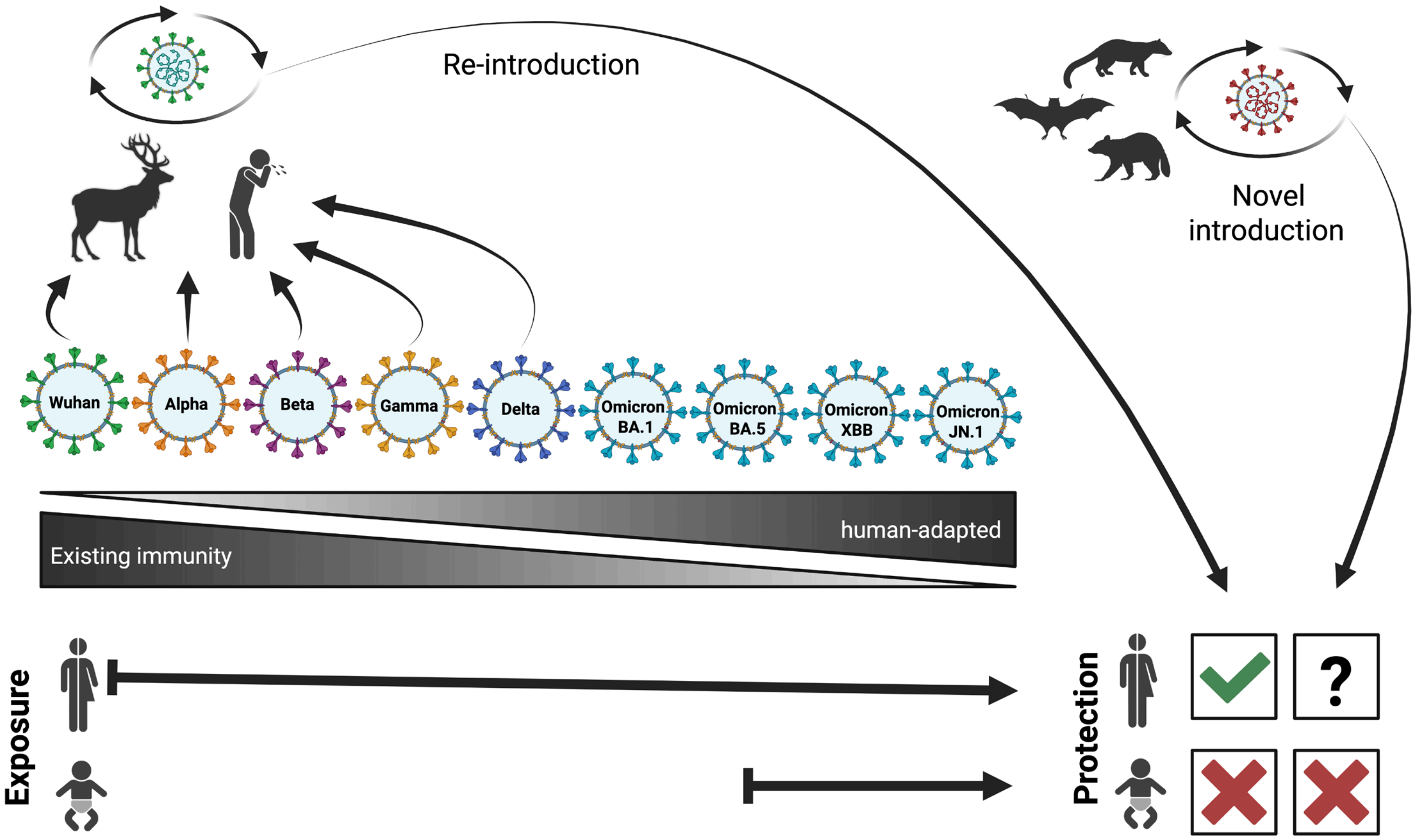

Figure S3. Immunity induced by natural infection or vaccine may protect adults from early SARS-CoV-2 VOCs but not children who were exposed to later VOCs and variant vaccines

Variants of Concern of SARS-CoV-2 have shown increasing mutations specifically within the spike protein. These mutations have enabled increased fitness for the viral receptor, human ACE2. Additionally, mutations have also occurred with increasing frequency within antigenically important sites such as the RBD, enabling escape from vaccine and natural infection induced immunity. Over time, older adults who have been vaccinated or exposed to previously circulating variants should exhibit some level of neutralizing antibody protection from these ancestral strains of SARS-CoV-2, should they re-emerge due to spillback from zoonotic reservoirs or from immunocompromised patients. Young children who were not exposed to earlier variants would likely lack effective immune responses should these viruses re-emerge. In the case of novel introductions of coronaviruses from zoonotic pools, such as bats, adults may have some level of protection should sufficient similarity exist between important antigenic domains due to previously stimulated immune responses from early SARS-CoV-2 ancestral strains.
