# Supplementary material for: Mouse-adapted SARS-CoV-2 Omicron BA.5 infection induces post-acute lung fibrosis in BALB/c mice
Source: J Virol. 2025 Nov 6;99(11):e01406-25. doi: 10.1128/jvi.01406-25 (PMC12645932; doi:10.1128/jvi.01406-25)
Supplement: Table S1 — Electron microscopy capture statistics. [file jvi.01406-25-s0004.pdf]

Table S1. Negative-stain EM data collection statistics

		SARS-CoV-2 BA.2 spike in complex with COV2-3605 Fab	SARS CoV-2 BA.2 spike in complex with COV2-3678 Fab
Data Deposition	EMDB		
Microscope setting	Microscope	TF-20	TF-20
	Voltage (kV)	200	200
	Detector	US-4000 CCD	US-4000 CCD
	Mag	x50000	x50000
	Pixel size	2.18	2.18
	Exposure (e-/Å^2)	30	30
	Defocus range (μm)	1-1.5	1-1.5
Data	# Micrographs	245	257
	# particles	21,545	10,341
	# particle after 2D	14,083	5,335
	Final particles #	4,978	3,074
	Symmetry	C1	C1
Model docking	Fab	PDB: 12E8	PDB: 12E8
